# Current Clinical Applications of *In Vivo* Gene Therapy with AAVs

**DOI:** 10.1016/j.ymthe.2020.12.007

**Published:** 2020-12-10

**Authors:** Jerry R. Mendell, Samiah A. Al-Zaidy, Louise R. Rodino-Klapac, Kimberly Goodspeed, Steven J. Gray, Christine N. Kay, Sanford L. Boye, Shannon E. Boye, Lindsey A. George, Stephanie Salabarria, Manuela Corti, Barry J. Byrne, Jacques P. Tremblay

**Affiliations:** 1 Center of Gene Therapy, Abigail Wexner Research Institute, Nationwide Children’s Hospital, Columbus, OH, USA; 2 Department of Pediatrics and Neurology, The Ohio State University, Columbus, OH, USA; 3 Al-Zaidy and Associates, LLC, Columbus, OH, USA; 4 Sarepta Therapeutics, Inc., Cambridge, MA, USA; 5 Department of Pediatrics, UT Southwestern Medical Center, Dallas, TX, USA; 6 Vitreoretinal Associates, Gainesville, FL, USA; 7 Department of Pediatrics, Powell Gene Therapy Center, University of Florida, Gainesville, FL, USA; 8 Division of Cellular and Molecular Therapeutics, University of Florida, Gainesville, FL, USA; 9 Division of Hematology and the Perelman Center for Cellular and Molecular Therapeutics, Philadelphia, PA, USA; 10 Children’s Hospital of Philadelphia, Philadelphia, PA, USA; 11 Department of Pediatrics, Perelman School of Medicine at the University of Pennsylvania, Philadelphia, PA, USA; 12 Department of Pediatrics, College of Medicine, University of Florida, Gainesville, FL, USA; 13 Powell Gene Therapy Center, University of Florida, Gainesville, FL, USA; 14 Centre de Recherche du CHUQ-Université Laval, Québec, QC, Canada

## Abstract

Hereditary diseases are caused by mutations in genes, and more than 7,000 rare diseases affect over 30 million Americans. For more than 30 years, hundreds of researchers have maintained that genetic modifications would provide effective treatments for many inherited human diseases, offering durable and possibly curative clinical benefit with a single treatment. This review is limited to gene therapy using adeno-associated virus (AAV) because the gene delivered by this vector does not integrate into the patient genome and has a low immunogenicity. There are now five treatments approved for commercialization and currently available, i.e., Luxturna, Zolgensma, the two chimeric antigen receptor T cell (CAR-T) therapies (Yescarta and Kymriah), and Strimvelis (the gammaretrovirus approved for adenosine deaminase-severe combined immunodeficiency [ADA-SCID] in Europe). Dozens of other treatments are under clinical trials. The review article presents a broad overview of the field of therapy by *in vivo* gene transfer. We review gene therapy for neuromuscular disorders (spinal muscular atrophy [SMA]; Duchenne muscular dystrophy [DMD]; X-linked myotubular myopathy [XLMTM]; and diseases of the central nervous system, including Alzheimer’s disease, Parkinson’s disease, Canavan disease, aromatic l-amino acid decarboxylase [AADC] deficiency, and giant axonal neuropathy), ocular disorders (Leber congenital amaurosis, age-related macular degeneration [AMD], choroideremia, achromatopsia, retinitis pigmentosa, and X-linked retinoschisis), the bleeding disorder hemophilia, and lysosomal storage disorders.

## Graphical Abstract

**Figure:**
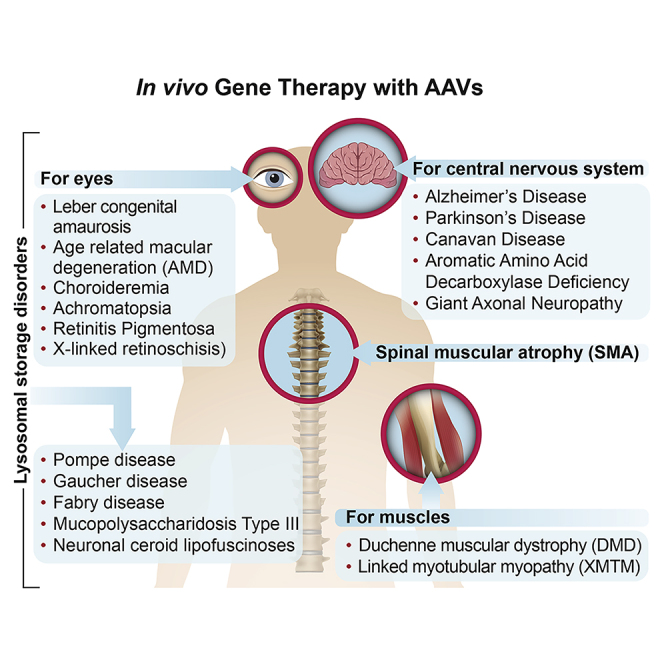

## Teaser

Researchers have been trying to develop therapies for hereditary diseases for more than 30 years. Several problems have been successfully resolved and thus Tremblay and colleagues are describing the recent progress of clinical applications of gene therapy. There are now several *in vivo* treatments under trials, and some approved for commercialization.

## Main Text

Hereditary diseases are caused by mutations in genes. There are more than 7,000 rare diseases affecting 30 million Americans, i.e., about 10% of the population. There are several hundred million patients around the world, according to the National Organization for Rare Disorders. Two-thirds of the patients are children. Currently, there are no effective therapies for more than 95 percent of these patients. The few drug-based treatments approved for genetic diseases at best manage or modify symptoms. However, they do not address the underlying genetic cause of the disease. Thus, these drugs must be administered for life.

Hundreds of researchers have dedicated their life to the pursuit of what initially appeared as an impossible dream: the development of gene therapies for hereditary diseases, i.e., a one-time curative repair or change to an individual’s affected gene that minimizes or even eliminates the symptoms for the entire life of the patient. This dream is now a reality: gene therapy greatly improves the outlook for currently incurable hereditary diseases!

This review is limited to gene therapy using adeno-associated virus (AAV) because the gene delivered by this vector does not integrate into the patient genome.

Glybera was approved by the US Food and Drug Administration (FDA) in October 2012 as the first AAV-mediated gene therapy to reach this milestone. Glybera corrected hereditary lipoprotein lipase deficiency (LPLD), which manifests as pancreatitis, recurrent abdominal pain, and eruptive fat-filled spots that result from very high triglyceride levels. However, the rarity of the disease (1 per million), the cost to the patient, and the expense to maintain therapeutic readiness by the company made it very difficult to continue gene delivery commercially. This form of gene therapy was no longer made available after 2018, at which time, only 31 people in the world had been treated.

There are now five treatments approved for commercialization and are currently available, i.e., Luxturna, Zolgensma, the two chimeric antigen receptor T cell (CAR-T) therapies (Yescarta and Kymriah), and Strimvelis (the gammaretrovirus approved for adenosine deaminase-severe combined immunodeficiency [ADA-SCID] in Europe). Dozens of other treatments are under clinical trials. The review article presents a broad overview of the field of therapy by *in vivo* gene transfer, which is based on direct administration of a gene-therapy vector to the body rather than transplant of gene-corrected cells. Herein, we will review *in vivo* gene therapy for neuromuscular disorders (spinal muscular atrophy [SMA]; Duchenne muscular dystrophy [DMD]; X-linked myotubular myopathy [XLMTM]; and diseases of the central nervous system [CNS], including Alzheimer’s disease [AD], Parkinson’s disease [PD], Canavan disease [CD], aromatic l-amino acid decarboxylase [AADC] deficiency, and giant axonal neuropathy [GAN]), ocular disorders (Leber congenital amaurosis [LCA], age-related macular degeneration [AMD], choroideremia, achromatopsia [ACHM], retinitis pigmentosa, and X-linked retinoschisis [XLRS]), the bleeding disorder hemophilia, and lysosomal storage disorders (LSDs). In each of these fields, the progress is fantastic, clinical trials are underway, and in some cases, the treatments are approved by regulatory agencies and commercialized.

### Clinical Gene Therapy in Neuromuscular Disorders

Clinical gene therapy in its various forms is rapidly evolving, offering the glimpse of hope that the broader community of rare disorders has long awaited. The most promising viral vector for gene transfer for neuromuscular diseases is AAV, having an excellent safety profile and efficiency in translation. We herein review three promising clinical AAV gene-therapy approaches that show promise for patients with severe and debilitating neuromuscular diseases.

#### SMA

SMA is a devastating neurodegenerative disease resulting from progressive loss of motor neurons.^1^ This autosomal recessive disorder results from a mutation in the survival motor neuron *SMN1* gene with an incidence of approximately 1:10,000 live births, 60% of whom have SMA type 1.^2^^,^^3^ The human *SMN* gene is an inverted duplication on chromosome 5q13.2. *SMN1* is telomeric and the highly homologous *SMN2*, lying in a centromeric position.^1^^,^^4^ An exon 7 point mutation of SMN2 gene results in exon splicing and exclusion from the final transcript, resulting in an unstable degradable protein.^5^ A full-length functional SMN protein is primarily the responsibility of *SMN1* with a small contribution from *SMN2*.^6^ In the absence of *SMN1*, the *SMN2* copy number is the major determinant of the clinical phenotype.^7^ Affected infants with 2 copies of *SMN2* are likely to develop severe type 1 (SMA1), characterized by rapidly progressive weakness, inability to sit independently, respiratory insufficiency with progression to death, or permanent ventilation prior to age 2.^8^

In preparation for human trials, a major step was demonstrating that AAV9 reached the nerve cells of the brainstem and spinal cord. It was confirmed in SMA murine models that systemically delivered self-complementary AAV9 (scAAV9) crossed the blood brain barrier and achieved high levels of neuronal transduction.^9^^,^^10^ Preclinical studies, delivering scAAV9-SMN to SMA pups, demonstrated positive effects on survival, growth, and neuromuscular transmission.^11^^,^^12^ Two key observations from these studies worth noting were both time of intervention and dose response effects on survival. Early treatment at postnatal day 2 extended the lifespan from 15 days to >250 days.^11^ The investigational new drug (IND) provided for an open-label, dose-ascending clinical gene-therapy trial starting May 13, 2014. A dose-ranging study of scAAV9.chicken b-actin (CB).SMN (START trial) at low (n = 3; 6.7 × 10^13^ vg/kg) and high (n = 6; 3.3 × 10^14^ vg/kg) dose was approved.^13^

Enrollment included symptomatic SMA1 infants with 2 *SMN2* copies and onset of symptoms prior to 6 months of age in the absence of permanent ventilation. At trial start, prednisolone was not included in the protocol. On day 9 after *SMN* gene delivery to the first patient, serum chemistries showed the alanine aminotransferase (ALT) increased 16× > normal. Based on these findings, protocol amendments were submitted to the FDA and included prednisone, 1 mg/kg per day begun 24 h prior to gene delivery and as a safety measure, reduced viral load for high dose from 3.3 × 10^14^ vg/kg to 2.0 × 10^14^ vg/kg. This is now considered a “therapeutic dose” and adopted for other gene-therapy clinical trials.

**Figure 1:**
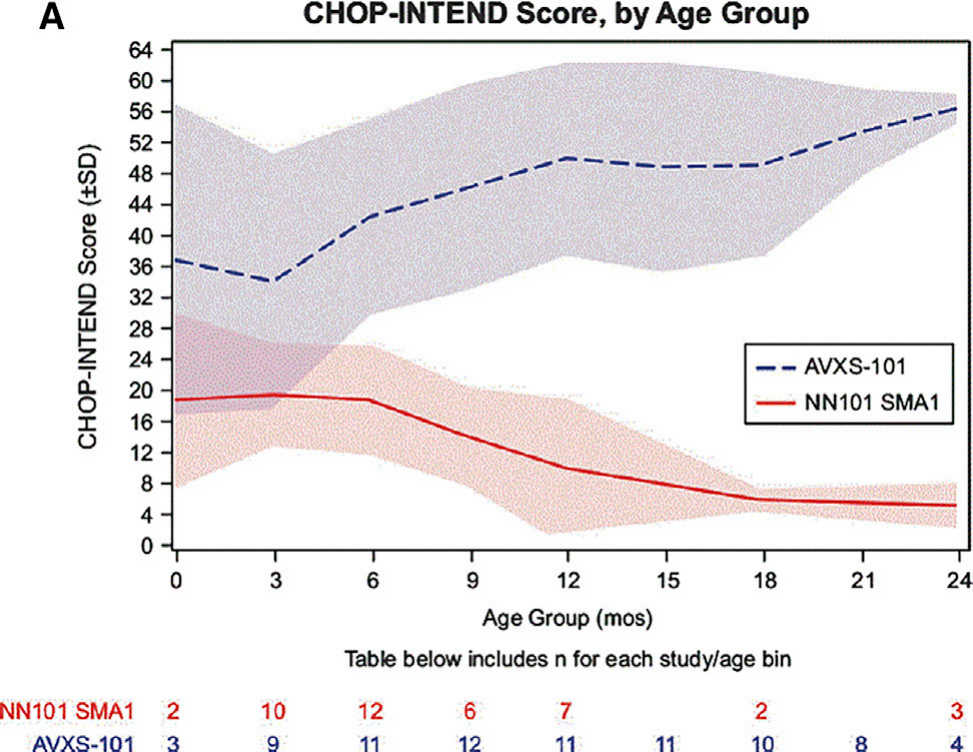
Maximum Longitudinal CHOP-INTEND Scores Reached for AVXS-101 Treated with the Therapeutic Dose Compared to the Prospective Natural History (NN101) Cohort Mean CHOP-INTEND scores by infant age are shown; shaded areas indicate the standard deviation for each mean at each study visit. (Reprinted from *Journal of Neuromuscular Diseases*^16^https://dx.doi.org/10.3233/JND-190403.

The SMA gene-therapy trial results perhaps exceeded expectations for the 15 SMA subjects enrolled (low dose n = 3; high dose n = 12). At study conclusion (December 2017), all infants were alive, and all 12 patients treated with the therapeutic dose were free of permanent ventilation.^14^ Treatment benefit was rapid: Children’s Hospital of Philadelphia Infant Test of Neuromuscular Disorders (CHOP-INTEND) increased 9.8 points at 1 month and 15.4 points at 3 months. In the therapeutic dose, 11 sat unassisted, 9 rolled over, 11 fed orally and could speak, and 2 woke independently.^13^ Figure 1 shows mean CHOP-INTEND scores for 24 months for SMA patients treated at the therapeutic dose compared to natural history. These motor milestones are unprecedented in SMA1 infants and were a clear indication of robust widespread transduction of the motor neurons. A key clinical observation that distinguished patients treated with therapeutic dose was preservation and recovery of respiratory and oral motor skills. Efficacy correlated with early treatment and high CHOP-INTEND.^15^ The single patient treated at 7.9 months did not respond to therapy. Elevated serum aminotransferases were seen in 4 patients and were attenuated by corticosteroids.

In April 2018, Novartis entered an agreement to acquire AveXis and continued following the 15 subjects enrolled in the study. In May 2019, onasemnogene abeparvovec (Zolgensma) received FDA approval as the first-ever systemically delivered AAV gene therapy. Following approval, noteworthy events include the continued long-term monitoring of subjects enrolled in the START trial. In the last data cut December 31, 2019, 11 of 12 patients treated in the first trial at the therapeutic dose survived without need for permanent ventilation. New milestones were documented, and the CHOP-INTEND score increased by 24.5 points. Two additional patients gained the milestone of standing with assistance. At data cut, the oldest patient receiving the therapeutic dose was 5.6 years of age, now 5.2 years since gene transfer.

**Figure 2:**
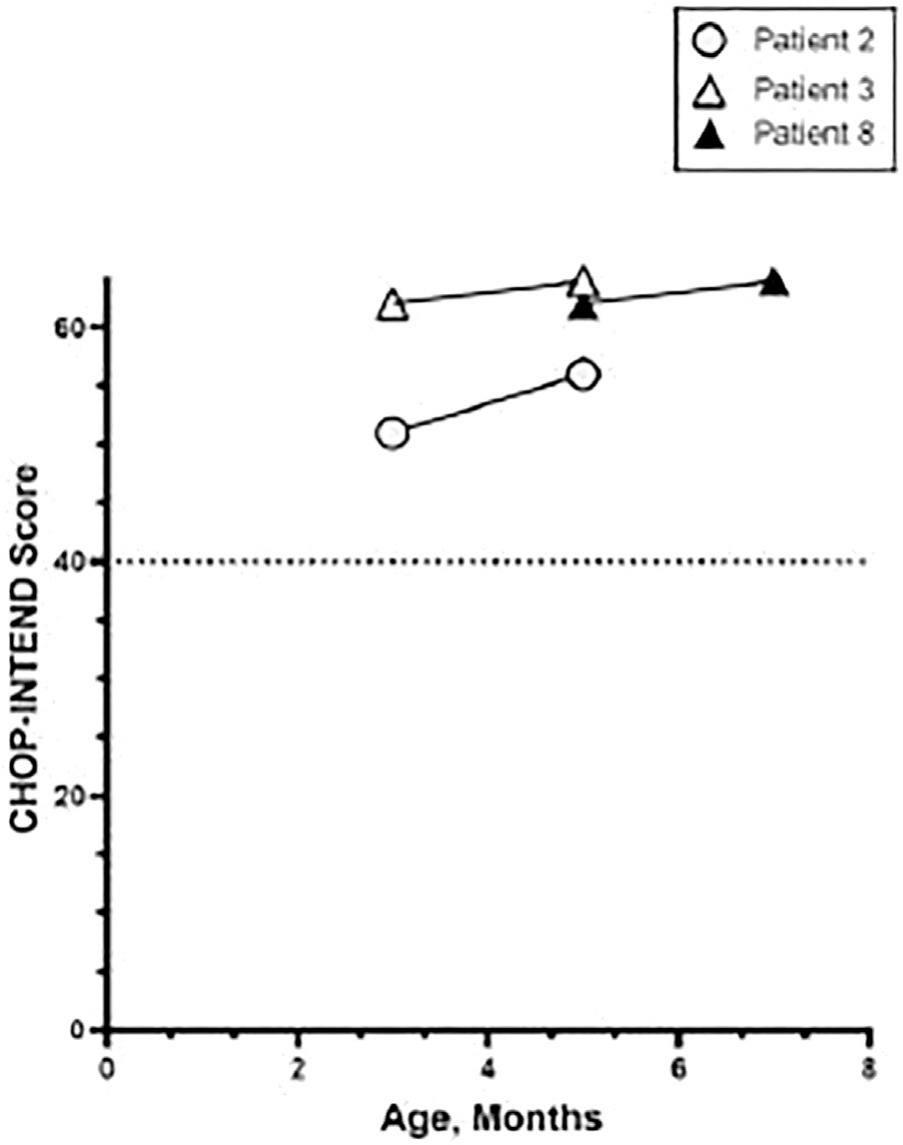
SMA Infants Recognized from Ohio Newborn Screening Receiving Gene Therapy Prior to Onset of Symptoms with High CHOP-INTEND Scores at Baseline Continued to Improve over Time Published in *Pediatrics*.^17^

To further assess the impact of early intervention, a new multicenter study of presymptomatic infants (≤6 weeks of age) with 2 or 3 copies of *SMN2* has begun (SPR1NT). Treatment at a mean age of 20 days shows improvement in CHOP-INTEND scores >50 in all subjects with survival up to 18 months (Figure 2). In 2019, SMA was added to the Recommended Uniform Screening Panel (RUSP), and numerous states have adopted this policymaking gene therapy available to newborn infants within the first few weeks after birth. In Ohio, 5 presymptomatic patients have been treated with onasemnogene abeparvovec, with the oldest now 12 months of age. The future looks bright for SMA patients.

The success of SMA type 1 gene therapy was extended to SMA patients ≥6 months to <5 years with three copies of SMN2, and eligibility required the ability to sit independently in the absence of walking without support. Three doses of scAAV.CB.SMN were to be administered intrathecally. Low- and mid-dose cohorts completed enrollment, but the high dose was put on partial hold by the FDA. This was in response to an AveXis reported preclinical study in nonhuman primates (NHPs) receiving intrathecal vector. They found dorsal root ganglia (DRG) mononuclear cell inflammation, sometimes accompanied by neuronal cell body degeneration or loss. The hold did not affect intravenous Zolgensma clinical trials.

Recent studies from the Wilson lab^18^ provide some further insight. Upon AAV vector infusion into the subarachnoid space in NHPs, pathology in the DRG is a consistent finding in virtually all animals. Similar findings have been seen, even in some studies when higher doses are administered systemically. However, in these NHPs, there is a notable absence of any clinical sequelae while using therapeutic transgenes. Thus, in the context of risk benefit, the response to gene delivery in clinical SMA disease^13^ outweighs concerns of the inflammatory infiltrate seen in DRG.

#### DMD

DMD is an X-linked, degenerative muscular dystrophy caused by mutations in the *DMD* gene.^19^ The gene product, dystrophin, is an integral cytoskeletal protein, anchoring the contractile actin filaments to the sarcolemma of both skeletal and smooth muscle cells. Over the course of the disease, loss of dystrophin results in membrane fragility, cycles of necrosis and regeneration, diminished regenerative capacity, and muscle replacement by fibrosis.^20^ Clinically, progressive muscle weakness results in loss of ambulation at 9−14 years of age and cardio-respiratory insufficiency leading to death in the second or third decade.^21^ Elevation of muscle creatine kinase (CK) indicative of ongoing dystrophic pathology is evident at birth.^22^ CK declines in nonambulatory patients as skeletal muscle is replaced by fibrosis.

The large size of the *DMD* gene renders it susceptible to spontaneous mutations. The incidence is 1:5,000 male births.^22^^,^^23^ Functional dystrophin production of a varying amount results from targeting specific mutations amenable to exon-skipping.^24^ Although some antisense oligonucleotide (AON) therapies have FDA approval,^25^ the applicable patient population and preservation of function remain limited. Gene replacement therapy is potentially an improved strategy for targeting a broader cohort of DMD patients. A major hurdle has been packaging the large DMD gene in AAV vectors limited to <5 kb. To circumvent this challenge, designs for miniaturizing the dystrophin gene have been developed. The adoption of a truncated version of dystrophin as a possible treatment for DMD is based on a Becker muscular dystrophy patient who remained ambulatory for seven decades despite a deletion of nearly half his *DMD* gene.^26^ Currently, transgenes incorporating diverse numbers of spectrin repeats (SRs) and hinges and delivery using different AAV serotypes are undergoing assessment of safety and efficacy in simultaneous systemic gene-therapy trials (Table 1) sponsored by Sarepta Therapeutics, Pfizer, and Solid Biosciences, summarized below.

**Table 1:** Miniaturizing the Dystrophin Gene for Adaptation for Clinical Trials for DMD

Company	Actin Binding Domain	Hinges	Rod Domain Spectrin Repeats	Hinges	Full Cystein-Rich Domain Required
Sarepta	N-term	1, 2, 4	spectrin repeats	1, 2, 3, 24	cysteine-rich domain
Pfizer	N-term	1, 3, 4	spectrin repeats	1, 2, 23, 24	cysteine-rich domain
Solid	N-term	1, 4	spectrin repeats	1,16,17, 24	cysteine-rich domain

Sarepta Therapeutics has recently reported the results of its phase 1/2 open-label, safety, and tolerability trial, conducted at Nationwide Children’s Hospital (ClinicalTrials.gov: NCT03375164).^27^ Enrollment included 4 DMD boys, mean age 4.8 years, with mutations between exons 18 and 58, taking prednisolone for ≥12 weeks, who received rAAVrh74.MHCK7.micro-dystrophin (SRP-9001) delivered through an extremity vein. Subjects with AAVrh74 total binding antibody titers >1:400 were excluded. The protocol included a single dose of daily prednisone, 1 mg/kg, starting 1 day prior to gene delivery, and continuing for 30 days (or more if needed). A single dose of SRP-9001, 2.0 × 10^14^ vg/kg was infused. The AAVrh74 serotype in combination with the tissue-specific MHCK7 promoter with an α-myosin enhancer predicted high levels of expression in both skeletal and cardiac muscle. The micro-dystrophin transgene contains SRs 1−3 that bind to the sarcolemma leading to improvement in sarcolemmal binding and force production, as well as SR 24 and hinges 1, 2, and 4.^28^ At 12 weeks, a gastrocnemius muscle biopsy with comparison to baseline showed a mean micro-dystrophin expression of 81.2% of muscle fibers, with mean intensity of 96%. Western blot (WB) showed a mean expression of 74.3% without adjustment for fat or fibrosis and 95.8% with adjustment. Serum CK remained decreased in all subjects (range 46%−85%) with functional improvement by a mean 5.5 points in the North Star Ambulatory Assessment (NSAA) at 1 year. There was also improvement in time to climb 4 stairs and run/walk 100. Histological findings revealed absent central nucleation and ring myofibers and reduction in percentage of collagen content in muscle post-treatment compared with baseline (mean 26.7% ± 8.4%).

The adverse event (AE) profile was minimal. Three patients had transient elevation of γ-glutamyltransferase that resolved with corticosteroids. A total 18 events were considered treatment related; most common was vomiting (50%). The cause for vomiting is unclear but did not correlate with AAV immunity. The safety and efficacy profile encouraged long-term follow-up and an ongoing phase 2, randomized, placebo-controlled, 96-week extension study with larger sample size (ClinicalTrials.gov: NCT03769116).

Pfizer pharmaceutical company provided results of its ongoing safety and tolerability trial of PF-06939926 at the recent American Society of Gene and Cell Therapy (ASGCT) meeting, May 15, 2020. rAAV9.mini-dystrohin was delivered to 9 DMD boys, ages 6.2 to 12.8; all were taking daily glucocorticoids, without pre-existing neutralizing AAV9 antibodies. The dose-ascending schedule included 1.0 × 10^14^ vg/kg (n = 3) or 3.0 × 10^14^ vg/kg (n = 6). The muscle biopsies at baseline compared mini-dystrophin to normal using liquid chromatography mass spectrometry (LCMS): 2 months 20% and at 12 months 24% (n = 3). At high dose, expression was 35% (n = 6) and 52% (n = 3), months 2 and 12, respectively. Immunofluorescence (IF) results were in the same range for samples tested at high and low dose with a slight decrease at 12 months. The NSAA for 3 subjects at 1 year showed a median increase of 3.5 points from baseline. The fat fraction estimated by MRI at 12 months showed a reduction of 8% at high dose (n = 3) and was unchanged at low dose.

The AE profile was more complex. More than 40% of participants experienced vomiting, nausea, decreased appetite, and pyrexia. Because of the report of acute kidney injury involving atypical hemolytic uremic syndrome (aHUS)-like with complement activation requiring hemodialysis and eculizumab, the trial was originally put on hold by Pfizer to enable protocol amendments (June 2019). In another subject, thrombocytopenia with aHUS-like complement activation required platelet transfusion and eculizumab. Pfizer has announced a planned phase 3, randomized, multicenter, double-blind, placebo-controlled trial inclusive of 99 subjects (C3391003) registered in ClinicalTrials.gov: NCT04281485.

Solid Biosciences using SGT-001 (ClinicalTrials.gov: NCT03368742) initiated a phase 1/2 open-label clinical trial using AAV9 and a CK8 muscle-specific promoter (SGT-001) targeting skeletal and cardiac muscle, the IGNITE DMD study. This cassette includes the neuronal nitric oxide synthase (nNOS)-binding domain, encoded by SRs 16/17, to enhance muscle perfusion.^29^^,^^30^ Six subjects have been enrolled and treated to date at the time of this review: low dose (5.0 × 10^13^ vg/kg, n = 3) and high dose (2.0 × 10^14^ vg/kg, n = 3). There have been repeated problems with complement activation resulting in 2 clinical holds by the FDA. The first in 2018 was related to thrombocytopenia. The second was in October 2019 because of more widespread complement activation affecting red blood cells (RBCs) and causing renal damage and cardiopulmonary insufficiency. Clinical results for the Solid trial were reported from the first cohort treated at low dose. In a single patient, microdystrophin was detected via WB below the 5% level of quantification and via IF in approximately 10% of fibers. Colocalization of nNOS and beta-sarcoglycan were also reported. In the other two subjects, microdystrophin was detected at very minimal levels by IF and none by WB. In March 2020, it was announced that 3 months post-treatment, the third patient, dosed at 2 × 10^14^ vg/kg, revealed 50%–70% of muscle fibers expressed micro-dystrophin, with WB detection at 8% of normal. The clinical hold for the Solid trial was lifted as of October 1, 2020.

#### XLMTM

The XLMTM is caused by mutations in the myotubularin 1 (*MTM1*) gene, located at Xq28.^31^ Myotubularin, a ubiquitously expressed phosphoinositide phosphatase, functions as a membrane enzyme and plays a role in skeletal muscle development and homeostasis.^32^ Mutations in the *MTM1*, resulting in loss of function, impact the excitation-coupling contraction mechanisms by disrupting the function and organization of the T-tubule network.^33^ Severe XLMTM, the most common form, presents at birth with hypotonia, external ophthalmoplegia, skeletal muscle weakness, and respiratory insufficiency.^34^ Signs of antenatal onset include reduced fetal movements and polyhydramnios. The muscle biopsy shows a uniform appearance of small muscle fibers with large centrally placed nuclei. The RECENSUS natural history study reported nearly all patients requiring respiratory support at birth with a 64% mortality at ≤18 months of age. Approximately 74% of patients surviving >18 months require tracheostomy and mechanical ventilation.^34^

Disruption of *Mtm1* in mice resembles human XLMTM, with similar pathology and early mortality.^35^ Intramuscular injections of the *Mtm1* delivered by AAV rescued muscle function, indicating that restoration of functional myotubularin could ameliorate the disease phenotype.^36^ Subsequently a series of studies from the Childers’ lab^37^, ^38^, ^39^ showed long-term therapeutic efficacy using systemic administration of an AAV8 vector expressing *Mtm1* using the muscle-specific desmin (DES) promoter. In the *Mtm1*-deficient mice, rAAV8.desmin.*Mtm1*, at 3 × 10^13^ vg/kg corrected muscle pathology and prolonged survival throughout the 6-month study. Low dose was less effective in the mouse. The canine model carrying the *MTM1* mutation improved function from locoregional vascular delivery. These studies prepared the way for a more extensive study in the canine model with whole-body correction of myotubular myopathy.^39^ In a dose-escalating intravenous study of rAAV8.desmin.cMTM1 (0.3 × 10^14^, 2 × 10^14^, and 5 × 10^14^ vg/kg) in the canine model (n = 3 per dose) at 10 weeks of age, with the comparison of saline-treated, age-matched mutants and normal littermates as controls, the two higher doses led to a reversal of the disease with clinical findings indistinguishable from normal without additional safety concerns. Together, these preclinical studies showed the feasibility, safety, and efficacy of gene therapy with AAV8 for long-term correction of XLMTM.^37^^,^^38^

The ASPIRO trial is a phase 1/2 open-label, randomized, ascending-dose study evaluating the safety and efficacy of AT132 (resamirigene bilparvovec). In this two-part gene-transfer study, a single intravenous dose is delivered in part 1, assessing two doses for safety and efficacy: 1 × 10^14^ vg/kg and 3 × 10^14^ vg/kg (ClinicalTrials.gov: NCT03199469). In part 2 of the study, eight subjects are randomized to either a treatment arm at 3 × 10^14^ vg/kg or to a delayed treatment control arm. As of August 2019, 12 patients were enrolled in the study with six (cohort 1) receiving low dose and cohort 2 (n = 4) treated at high dose compared to untreated controls. Preliminary results shared by Audentes showed a favorable response in safety and efficacy and follow-up ranging between 4 and 72 weeks. All treated subjects but one had a clinically meaningful improvement in respiratory function, with a reduction in daily hours of ventilatory requirement as well as improvement in the mean inspiratory pressure (MIP). A positive response in motor function measured by CHOP-INTEND was also seen. Muscle biopsies available for 9 subjects demonstrated robust dose-dependent tissue transduction and myotubularin protein expression with an improvement in overall muscle histology. This contrasts with natural history data wherein XLMTM subjects had a 2.7-point annual decline from baseline in CHOP-INTEND scores and a reduction in MIP over 12 months.^40^ In 2018, following an acknowledgment of the progress made in this trial, the FDA granted AT132 the regenerative medicine advanced therapy (RMAT) designation, equivalent to FDA’s Fast Track and Breakthrough Therapy.

Unfortunately, as the trial appeared to be proceeding in a favorable manner, in May 2020, Audentes, the trial sponsor, reported in a letter to patient groups that a participant in the trial died after receiving AT132 at the higher dose of 3 × 10^14^ vg/kg. Subsequently, in June 2020, Audentes shared further details when a second of 3 in the high-dose cohort also died. The two deaths followed a similar clinical course, each suffering from progressive liver disease 4−6 weeks after receiving the gene therapy. The direct cause of death was generalized sepsis following the liver complications. None of the six patients who received the lower dose experienced serious AEs (SAEs), despite four of them having a history of hepatobiliary disease. Notable features among the three patients with these SAEs include older age, heavier weight, evidence of pre-existing hepatobiliary disease, and dosing with the higher dose of 3 × 10^14^ vg/kg. The XLMTM trial is now on clinical hold by the FDA and searching for further explanation. Active data collection is ongoing.

In conclusion, in this review, we highlight gene-therapy clinical trials in three neuromuscular disorders to illustrate the potential for the broader application of systemic delivery. Undoubtedly, significant advances have been made in systemic gene delivery; however, several limitations remain to be resolved. The efficacy and safety in each disease require further analysis and long-term follow-up. The severe complications in the XLMTM trial require further study.

### Gene Therapy for CNS Diseases

Gene therapy for CNS diseases using AAV vectors was initiated nearly 2 decades ago using stereotaxic intracerebral delivery of AAV2 vectors. Early pioneering clinical trials were for CD, AD, and PD. Whereas the approaches for AD and PD were focal bilateral injections, the clinical trial for CD attempted stereotaxic AAV2 administration to 12 distinct sites to maximize vector distribution throughout the brain. AADC deficiency disorder followed, again using AAV2, and was a pivotal demonstration of disease-altering efficacy in a CNS gene-therapy trial. These early CNS gene-therapy trials demonstrated the feasibility of AAV-based gene transfer to treat the CNS safely.

#### AD

AD is neurodegenerative disorder that is the leading cause of age-related dementia. A deep understanding of AD and the associated neuropathology have led to development of numerous viral-mediated gene-transfer approaches for AD, as reviewed by Raikwar et al.^41^ One approach that has entered clinical trials is delivery of nerve growth factor (NGF), which is hypothesized to promote survival of cholinergic neurons.^42^^,^^43^ A phase 1 study of bilateral intracerebral delivery AAV2-NGF to the basal forebrain of patients with mild to moderate AD-associated dementia showed promising results. The surgical delivery was safe and well tolerated, and there was lack of clinically relevant progression of disease 2 years postinjection.^44^ However, in the subsequent randomized controlled phase 2 study (n = 49), efficacy endpoints were not met.^45^ Despite this early failure, there are numerous additional applications in the gene-therapy pipeline for AD.

#### PD

PD is a neurodegenerative movement disorder characterized by bradykinesia, gait impairment, and later cognitive decline that is caused by a loss of dopaminergic neurons in the basal ganglia. Viral-mediated gene-therapy approaches aimed at modulating GABAergic neuronal signaling (AAV2-GAD [AAV2-glutamic acid decarboxylase]) and increasing dopamine production (AAV2-hAADC, amino acid decarboxylase) have been studied in early-phase clinical trials.

The initial open-label, dose-escalation phase 1 study explored the delivery of AAV-GAD injected unilaterally into the subthalamic nucleus of 12 PD patients. There were no treatment-related AEs, and all subjects demonstrated improvements in motor functioning with diminished thalamic metabolisms within the treated hemisphere.^46^ In the phase 2 randomized controlled trial, patients with advanced PD received either a sham procedure (n = 23) or bilateral infusion of AAV2-GAD to the subthalamic nuclei (n = 22). The procedure was again well tolerated, and patients in the treatment group demonstrated improvement in motor function.^47^ These results were sustained out to 12 months postinjection.^48^ It is postulated that delivery of GAD to this brain region leads to formation of new functional pathways between the subthalamic neurons and motor cortical regions, termed “GAD-related pathways,” which correlate to clinical improvements in the treatment group.^49^

In an alternative approach, AADC, an enzyme involved in the synthesis of dopamine, is delivered to the putamen of PD patients, with the goal of increasing dopamine production. In a phase 1 study of bilateral intrastriatal infusion of AAV-hAADC in five patients with moderately advanced PD, there was a 30% increase in AADC expression in the putamen and a modest clinical improvement in patients.^50^ A subsequent dose-escalation study demonstrated similar results with a 30% increase in AADC expression on positron emission tomography (PET) scan in the low-dose cohort and 75% increase in the high-dose cohort.^51^ In this study, three patients had a postoperative intracranial hemorrhage (one symptomatic, two asymptomatic).^51^ In long-term follow-up, effects persisted for up to 96 weeks.^52^ Most recently, in a phase 1 gene-delivery optimization study, gadoteridol is coadministered with AAV2-AADC via MRI-guided infusion to the putamen to facilitate visualization of the vector spread and coverage. In this three-level dose-escalation study (n = 5 per dosing level), there was a dose-responsive increase in AADC activity ranging from 13% to 79% that correlated to improvements in motor outcomes, including increased response to levodopa without dyskinesia.^53^ Both approaches demonstrated favorable safety profiles and promising clinical response, but larger, well-controlled studies are needed.

#### CD

CD is a leukodystrophy caused by pathogenic variants of the aspartoacylase gene (ASPA). ASPA is expressed by oligodendrocytes and is responsible for the degradation of N-acetylaspartate (NAA) through deacetylation. Elevations of NAA in the CNS have variable downstream effects that may explain the underlying pathophysiology of CD, including aberrant myelination, parenchymal edema, and vacuolation of the white matter (reviewed by Leone et al.^54^). The clinical presentation of CD varies based on the residual activity levels of ASPA and corresponding concentrations of NAA. It is a neurodegenerative disorder with hydrocephalus due to progressive spongy neurodegeneration, progressive neurologic disability, intractable epilepsy, feeding intolerance, and premature death in adolescence or early adulthood. The AAV2 approach evolved from earlier studies of gene transfer using a lipid-entrapped polycation-condensed delivery system (LPD) in conjunction with AAV-based plasmids containing rASPA.^55^ Whereas this approach was well tolerated and led to both biochemical and clinical improvements, improved viral-mediated gene-transfer technologies using AAV2 were studied in a phase 1 study of intracranial infusions via six cranial burr holes in patients with CD (n = 13).^56^ The procedure was well tolerated with only minimal systemic inflammation and a demonstrated, promising long-term safety and clinical efficacy. Global CNS concentrations of NAA, as measured by magnetic resonance (MR) spectroscopy, were decreased after AAV2-ASPA delivery, especially in the basal ganglia. Similarly, the T1 relaxation time decreased in white-matter tracts, especially the splenium of the corpus callosum, and some patients demonstrated a halting or reversal of brain atrophy. Finally, improvement in these biochemical and imaging biomarkers was accompanied by stabilization to improvements in gross motor functioning, although social and cognitive recovery was less consistent. The reversal of biochemical and structural biomarkers suggests that the process is reversible; however, earlier intervention is suspected to lead to a more robust clinical response.^54^ New technologies using AAV-ASPA targeting oligodendrocytes are now being studied.^57^

#### AADC Deficiency

After promising results in the AAV2-AADC studies for PD, investigators sought to apply this technology to a rare neurogenetic disorder of childhood. AADC deficiency disorder is a rare inherited disorder of neurotransmitter synthesis caused by biallelic variants in the dopa decarboxylase (DDC) gene on chromosome 7.^58^ Although the clinical spectrum can vary, 80% of patients are classified as severe and present in infancy with hypotonia, growth retardation, and marked motor deficits. They never attain proper head control or the ability to sit independently and suffer from frequent episodes of dystonia and oculogyric crises. Autonomic dysfunction and severe emotional irritability are frequently reported.^58^ Individuals typically die by the age of 5 years old.^59^

In 2012, the initial gene-transfer compassionate-use clinical trial of AAV2/hAADC was performed in Taiwan. Hwu et al.^59^ selected four subjects with confirmed diagnoses of AADC from the twenty known living patients in Taiwan. On baseline assessment, all patients were bedridden, lacked head control, and were unable to speak. Oculogyric crises were reported every 2 to 3 days, and caregivers reported marked irritability, excessive sweating, and unstable body temperatures. Patients received AAV2/hAADC by direct bilateral intraputaminal injection and followed for up to 24 months. Transient dyskinesias sufficient to interfere with feeding were reported in two of four subjects, and one subject had significant apneic events that subsided within 10 months of dosing. All four subjects demonstrated improvements across both motor and cognitive developmental outcomes, and caregivers reported a decrease in the frequency and intensity of oculogyric crises, diminished irritability, and increased temperature stability. Further, biomarker data were compelling, demonstrating a 45% to 86% increase in uptake of dopamine on PET scan as compared to baseline measurements, and all demonstrated an increase in cerebrospinal fluid (CSF) levels of dopamine and serotonin metabolites.^59^

In the first phase 1/2, open-label clinical trial at the National Taiwan University Hospital (Taipei, Taiwan), an additional ten children, ages 24 months and up, with confirmed AADC deficiency diagnoses were dosed with intraputaminal AAV2-hAADC. One subject died from an unrelated cause, but the remaining survivors demonstrated remarkable improvements in motor functioning (increase of 62 points on the Peabody Developmental Motor Scale). Reported AEs included pyrexia and transient orofacial dyskinesia that resolved with risperidone.^60^ Similar results were found in a second open-label phase 1/2 study of a more genetically diverse population (n = 6).^61^ Within 2 months, all had improvement in their voluntary movements, two weaned off mechanical ventilation, four regained the ability to eat by mouth, and all showed improvements in dystonic episodes, irritability, and autonomic dysfunction.^61^

#### GAN

GAN is an autosomal recessive neurodegenerative disorder of the central and peripheral nervous system that typically presents with progressive weakness and ataxia. Individuals also suffer sensory loss and loss of ambulation and succumb to respiratory failure.^62^ Based on neuropathological studies, there is a loss of gigaxonin expression that affects the cerebellar cortex, brainstem, and posterior columns of the spinal cord, making it an ideal candidate disorder for intrathecal delivery of AAV9 gene transfer (reviewed by Bailey et al.^63^). Gigaxonin is required to organize and degrade intermediate filaments and leads to enlarged axons with densely bundled intermediate filaments (reviewed by Bailey et al.^63^). A phase 1 dose escalation study of intrathecal AAV9/GAN is underway at the National Institutes of Health (NIH; ClinicalTrials.gov: NCT02362438).

#### Future Outlook

Nearly 2 decades after the initial intracerebral gene-transfer trials using AAV2, methods for gene transfer to the CNS have greatly expanded. When focal gene transfer is desired, such as for PD and AADC, more accurate gene transfer to a greater volume of brain tissue can be achieved with AAV2 vectors using methods such as MRI-guided convection-enhanced delivery.^64^, ^65^, ^66^ However, most CNS disorders would ideally require broad and efficient gene transfer to the entire CNS. The discovery of newer AAV capsids, such as AAV9, have permitted a much wider degree of gene transfer than multiple stereotaxic injections.^9^^,^^10^^,^^67^, ^68^, ^69^ Aside from numerous studies in animal models, the application of AAV9 has been demonstrated in clinical trials after an intrathecal injection (as for GAN) or by an intravenous injection (as for SMA^13^). It is anticipated that the use of AAV9 or similar AAV capsids will broaden the application of gene therapy to more CNS disorders in the near future. However, whereas AAV9 has greatly expanded the ability to treat a larger number of CNS disorders, it still targets a minority of cells throughout the brain.^70^ Looking forward, a newer generation of AAV capsids with greater CNS targeting efficiency would increase the effectiveness of CNS-directed gene-therapy treatments, as well as expand the number of diseases that could potentially be treated with gene therapy.

### Clinical *In Vivo* Gene Therapy for Ocular Disorders

Gene therapy gained its place in mainstream medical practice following FDA approval of Luxturna, an AAV2- based treatment for the inherited retinal disease (IRD) retinal pigment epithelium (*RPE*)*65*-LCA (LCA2). This success was based on decades of work by multiple groups, one of which went on to commercialize the product.^71^, ^72^, ^73^, ^74^, ^75^, ^76^ Treated patients exhibited life-changing improvements in light sensitivity and visually guided behavior. Detailed summaries of RPE65 biology, preclinical studies in animal models, and the treatment of LCA2 with gene therapy by multiple groups are already published.^77^^,^^78^ The dramatic success of this program catalyzed academia and industry alike to establish proof of concept that gene therapy could restore or preserve vision in animal models of other retinal diseases, including, but not limited to, AMD, choroideremia, ACHM, retinitis pigmentosa, and XLRS.^79^, ^80^, ^81^, ^82^, ^83^, ^84^, ^85^ Interestingly, however, these preclinical successes have not consistently translated to clinical outcomes as robust as those observed in LCA2 patients.^86^, ^87^, ^88^, ^89^, ^90^, ^91^, ^92^ Whereas the reasons for this discrepancy have yet to be fully elucidated, insufficient transgene expression mediated by AAV in the target cells and/or immune response likely played a role. Future success in the retinal gene-therapy space and the broader gene-therapy field will depend on identifying feasible therapeutic dose ranges that are based on the proven ability to (1) target the appropriate cell type in a primate retina and (2) drive sufficient levels of therapeutic transgene expression. Whereas many factors contribute to AAV’s tropism, transduction efficiency, and associated immune response in retina, route of delivery is especially critical. A summary of current and developing approaches, their advantages and disadvantages, and relevant clinical examples are discussed below.

#### Subretinal Injection (SRI)

SRI is employed in the majority of clinical trials because it allows for placement of the therapeutic *in situ* (in a surgically created space between photoreceptors (PRs) and RPE referred to as a subretinal “bleb”). The majority of IRDs are caused by mutations in PR-specific genes. In addition to its proximity to the most common clinical target cells (i.e., RPE and PR), SRI is attractive because of this compartment’s immune privilege. Unlike systemically delivered AAV, subretinally delivered vectors elicit a relatively reduced immune response akin to anterior chamber-associated immune deviation (ACAID).^93^^,^^94^ However, SRI is a challenging technique, described as “almost a subspecialty unto itself.”^95^ It requires a vitrectomy (removal of vitreous humor) and retinotomy (passage of needle through the retina), which can be associated with complications, such as retinal tears, cataract progression, or retinal/choroidal hemmorage.^96^ Creation of the subretinal bleb requires detaching retina from the underlying RPE. The cone-exclusive fovea is especially sensitive to detachment. SRI of vector under the fovea of some LCA2 patients led to central retinal thinning and loss of visual acuity.^75^ Similar decreases in retinal thickness were observed in choroideremia patients.^96^

The surgical technique of subretinal gene therapy is predicated upon established subretinal procedures, such as subretinal tissue plasminogen activator (tPA) injection for subretinal hemorrhage associated with neovascular AMD.^96^ However, specific considerations must be made to adapt the technique to the particular characteristics of retinal structure present in IRDs. Vitreoretinal surgeons well versed in subretinal gene therapy have reported the utility of intraoperative optical coherence tomography (OCT), allowing *in vivo* real-time feedback during surgical cases.^97^ The creation of the subretinal bleb with a microneedle (typically a 38- to 41-gauge, Teflon-tipped, either extendable or nonextendable, cannula that is placed through a pars plana trocar) is a challenging and critical step in the procedure.^98^ The needle-penetration step has a narrow margin of error: excessively deep penetration of the needle tip can result in hemorrhage, cannula tip obstruction, unintentional suprachoroidal delivery of vector, or permanent RPE injury; however, too shallow needle penetration can create retinoschisis by intraretinal hydration during bleb formation.^97^ Some surgeons create a “pre-bleb” made with balanced salt solution (BSS) prior to the injection of the vector into this space, which may prevent loss of vector into the vitreous cavity during the bleb creation. Another surgical consideration is the inherent difficulty of uniform and accurate volume delivery associated with the current transvitreal subretinal delivery method. Vector volume can be affected by use of a BSS pre-bleb, loss of vector from vitreous egress from retinotomy site, and incomplete target vector volume delivery due to surgeon discretion (concern for foveal stretching or macular hole formation or other safety considerations). In clinical trials where dose-escalation decisions are being made with small numbers of subjects, it may be challenging to appropriately make safety or efficacy decisions unless confirmation of precise and uniform vector volume delivery can be achieved in all patients dosed.

It is also important to note that, despite its relative immune privilege, AAV vectors are still capable of reaching an adverse effect level and eliciting host-cell responses in the subretinal space. In phase I/II clinical trials for *RPE65*-LCA at University College London (ClinicalTrials.gov: NCT00643747), ocular inflammation was noted following SRI of 1 × 10^12^ vg of AAV2-RPE65.^71^^,^^99^ There was no inflammation noted in the University of Pennsylvania/University of Florida trial (ClinicalTrials.gov: NCT00481546). In the Nantes University Hospital trial (ClinicalTrials.gov: NCT01496040), which notably used a different AAV capsid (AAV4), inflammation was noted at 4.8 × 10^10^ vg.^100^ In Spark Therapeutics’ phase III trial (ClinicalTrials.gov: NCT00999609), mild inflammation was observed at 1.5 × 10^11^ vg.^76^^,^^101^ Significant inflammation was also observed at 1 × 10^11^ vg in clinical trials for choroideremia.^90^^,^^102^ The different doses at which inflammation has been observed clinically may be attributed to differences in vector production and characterization, AAV capsid, and underlying retinal disease state.

As focus has now shifted to evaluating gene therapies for IRDs where the target cells are PRs, it is worth considering the relationship between abundance of the gene-replacement product and vector dosing. For any gene therapy to be successful, sufficient therapeutic transgene expression (e.g., protein) levels must be achieved at doses that do not cause unmanageable inflammation. Ideally, a sufficient range between the minimum effective dose in animal models and the NOAEL (no observable adverse effect level) should exist such that a phase I/II dose-escalation study can be performed. Logic dictates IRDs caused by defects in retinal proteins expressed at relatively low levels may be more easily addressed by gene therapy. A comparison of preclinical findings in large animal models of IRD versus clinical outcomes supports this concept, although published clinical outcomes remain scant to date. Preclinical studies sponsored by AGTC evaluated AAV-RPGR (retinitis pigmentosa GTPase regulator) in the diseased canine model of RPGR X-linked retinitis pigmentosa (XLRP).^103^ Efficacy (improvements in fundus autofluorescence) was reported at doses as low as 1.8 × 10^9^ vg,^103^ and inflammation was not observed until a dose of 4.5 × 10^11^ vg.^103^ Biogen’s 6-month AAV-RPGR phase I/II clinical trial results were presented at this year’s ASGCT meeting. It reported improvements in the visual fields in six treated XLRP patients, “exceptional visual improvement,” and “evidence of possible outer segment regeneration” in one patient treated at the high dose (5 × 10^11^ vg). Manageable inflammation was only observed in this high-dose group. Whereas not yet presented/published, AGTC’s press release^104^ (date accessed 6-12-20) states that 9 out of 17 treated patients experienced improvements in vision, as measured by microperimetry and/or best-corrected visual acuity (BCVA) at 3 months postinjection. Only patients with submacular injections showed this improvement. At 6 months postinjection, improvements in those nine patients were stable. In four of these patients (4/8 tested), there were improvements in visual sensitivity (i.e., microperimetry). Proteomic analysis reveals there are approximately 2,000 molecules of RPGR per PR sensory cilium (PSC), making it the 1,087^th^ most enriched protein in rods.^105^ In contrast, preclinical studies evaluating AAV-cyclic nucleotide-gated channel subunit beta 3 (CNGB3) in a dog model of CNGB3 ACHM revealed only modest improvements in retinal function at 5 × 10^10^ vg and inflammation at 5 × 10^11^ vg. Phase I/II trials for *CNGB3* ACHM were initiated in 2016 by both AGTC and MeiraGTx, but clinical outcomes have not yet been presented/published. Cyclic nucleotide-gated channels are highly expressed (∼61,000 molecules per PSC), making them much more abundant than RPGR.^105^ Preclinical studies in a sheep model of cyclic nucleotide-gated channel subunit alpha 3 (CNGA3) ACHM revealed robust improvements in retinal function at 1.8 × 10^11^ vg (lowest dose tested) and inflammation at 6.0 × 10^11^ vg. Phase I/II clinical trials conducted at the University Hospital Tuebingen and Ludwig Maximilian University of Munich (ClinicalTrials.gov: NCT02610582) treated patients at the following doses: 1 × 10^10^ vg, 5 × 10^10^ vg, or 1 × 10^11^ vg.^91^ No unmanageable inflammation was reported, and modest improvements in visual acuity, contrast sensitivity, and chromatic discrimination thresholds were observed at all doses, consistent with biological activity mediated by vector.^92^ Doses required to confer therapy in preclinical studies were higher in *CNGA3*-ACHM relative to *RPGR*-XLRP, although the CNGA3-ACHM clinical results suggest that IRDs caused by defects in highly expressed genes may still be successful.

Some IRDs, such as *MYO7A*-associated Usher syndrome and *ABCA4*-associated Stargardt disease, are caused by mutations in genes for which coding sequences are too large to fit within a standard AAV vector (packaging capacity ∼5 kb). Lentivirus, which can accommodate a larger payload (9.7 kb), was therefore chosen to address these IRDs in phase I/II clinical trials that began in 2011/2012. No reports of biological activity have been published to date for either trial. This is thought to be attributed to the fact that lentivirus poorly transduces postmitotic PRs,^106^^,^^107^ the target cell in both USH1B and Stargardt. Efforts are currently underway by multiple groups to develop dual AAV vector platforms that will promote delivery of large genes to PRs.^108^^,^^109^

Beyond monogenic disease, AAV is also being used to vectorize anti-vascular endothelial growth factor (VEGF) reagents to the retina via SRI to serve as a one-time treatment for the neovascular form of AMD (wet AMD). This represents a potential improvement over the standard-of-care monthly intravitreal injections (IVIs) of VEGF inhibitors that can suffer from low compliance. REGENXBIO recently completed a phase I/IIa trial in which patients across five cohorts received doses of AAV8-anti VEGF fab between 3 × 10^9^ vg and 2.4 × 10^11^ vg per eye. Dose-dependent increases in protein expression levels were observed at 1 month p.i. There were no drug-related AEs and no clinical signs of an immune response or drug-related ocular inflammation. Whereas no clear signs of improvements were noted in cohort 1 (3 × 10^9^ vg/eye) or cohort 2 (1 × 10^10^ vg/eye), patients in cohort 3 (6 × 10^10^ vg/eye) showed improvements in mean BCVA and central retinal thickness. 50% of patients in cohort 3 remain injection free (i.e., no IVIs VEGF inhibitors) for up to 2 years post-treatment. Most recently, results from cohort 5 (2.5 × 10^11^ vg/eye) showed that 73% of patients remained injection free for at least 9 months post-treatment. REGENXBIO has future plans to modify its delivery approach. Its goal is to deliver AAV to the subretinal space via a microcannula that accesses the retina posteriorly from the suprachoroidal space (see section below) and in so doing, increase accessibility to the treatment (will not require vitrectomy/full-blown surgery).

Another topic of interest within the IRD gene-therapy field is the evolving analysis of risk to benefit for the subretinal approach. An example is whether detachment of the cone-exclusive fovea via SRI is advisable. In certain IRDs characterized by the presence of severe functional deficits in spite of retinal preservation (e.g., *GUCY2D*-LCA1), SRI of the macula poses an attractive risk:benefit ratio.^110^^,^^111^ Put simply, patients with completely dysfunctional foveal cones have less to risk in terms of function and a large potential for gain as their preserved retinal laminar architecture makes them (1) more likely to tolerate surgical foveal detachment and (2) potentially more receptive to therapy. This is in contrast with patients who retain cone function and have actively degenerating retina (i.e., retinitis pigmentosa). In the latter scenario, SRI of AAVs capable of laterally spreading beyond the margin of detachment may mediate therapeutic levels of gene expression in foveal cones while avoiding the risks associated with foveal detachment. Indeed, extra-foveal SRI of such vectors (AAV44.9 based) promoted 98% transduction of foveal cones in macaque without the need to detach the fovea during surgery.^112^ In addition, these novel vectors that spread laterally from the injection bleb may also enable functional improvements across a greater expanse of retina.^112^ Despite the surgical complexity, the many advantages that SRI offers (robust gene expression in outer retinal cells, delivery to a relatively immune-privileged site, its proven success in addressing IRDs, and novel capsid technologies being explored to further increase its safety) suggest that this delivery approach will continue to be used for some time.

#### Suprachoroidal Injection

Suprachoroidal injection is a relatively novel mode of ocular AAV delivery that is currently eliciting preclinical interest. Transscleral suprachoroidal viral delivery aims to achieve efficient outer retinal transduction in the absence of vitreoretinal surgery. The suprachoroidal space is a potential space between the choroid and scleral wall of the eye that can be accessed via surgical cannulation^113^ or transscleral microneedles.^114^^,^^115^ Data in rat, pig, and NHP suggest plausibility of transduction of both RPE and PRs via suprachoroidal AAV gene delivery, although results have been inconsistent across studies.^116^, ^117^, ^118^ A recent report showed that suprachoroidal AAV delivery produced transient expression restricted primarily to the RPE and local infiltration of inflammatory cells. In contrast, when the same novel device was used to subretinally deliver AAV8 (access from the suprachoroidal space followed by transversal of Bruch’s membrane with an extendable microcannula), focal transgene expression in RPE and PRs and minimal intraocular inflammation were observed.^117^ These devices have been evaluated in preclinical and clinical studies of cell-based therapies^119^ and are now being considered for delivery of AAV. Additional work is needed to refine the method and investigate associated local and systemic immune responses prior to moving this approach into the clinical arena.

#### IVI

IVI is by the far the least invasive delivery route under consideration for treating IRD and may protect inherently thin and degenerate retinas from additional mechanical damage potentially caused by vitrectomy and surgical subretinal detachment. However, it requires an AAV capsid capable of “penetrating” through the retina from the vitreous. One advantage of IVI is that the procedure can be performed in a clinic setting, thereby increasing accessibility of gene therapies to larger patient populations. However, the limited clinical data^88^^,^^89^ and NHP studies^120^ utilizing currently available capsids indicate that IVI AAVs contend with many barriers that may reduce gene expression and prevent therapeutic benefit in a majority of patients (i.e., dilution/neutralization in vitreous, inner-limiting membrane). Current AAVs in clinical testing mediate transduction of retinal ganglion cells (RGCs) in the macular “ring,” Müller glia, and sparse PRs adjacent to large blood vessels, some foveal cones, and non-neuronal cells of the ciliary body.^121^, ^122^, ^123^, ^124^ As such, it is not surprising that the only IVI-based clinical trials to demonstrate biological activity thus far have targeted RGCs in the inner retina either to provide direct benefit to these cells (*ND4*-Leber hereditary optic neuropathy [LHON])^125^^,^^126^ or utilize them as a depot for secretion of an anti-VEGF reagent (wet AMD).^88^

To date, there is no clinical evidence that an IVI-delivered AAV vector can transduce outer retinal cells (PRs/RPE) at sufficient levels to mediate a therapeutic response. Intravitreally delivered AAV8-*RS1* failed to restore retinal structure/function in XLRS patients (ClinicalTrials.gov: NCT02317887).^89^ Despite the secretory nature of the RS1 protein, studies performed in Rs1h knockout mice show that enduring therapy is achieved when AAV-mediated RS1 is produced within PRs.^127^ AAV8 does not efficiently transduce PRs following IVI of intact retina.^117^ The capsid choice in this trial was dictated by transduction observed in the XLRS mouse model and perhaps the expectation that the presence of schisis cavities in XLRS patient retinas would facilitate transduction.^128^ Taken together, the lack of efficacy in this trial was at least partially due to inefficient targeting of *RS1* to PRs. Immune response also played an important role. The vitreous is not as immune privileged as the subretinal space.^129^ When comparing the biodistribution of AAV following IVI versus SRI, IVI results in relatively more systemic exposure.^130^^,^^131^ Similar to SRI, inflammation caused by IVI AAV appears to be dose dependent. IVI of AAV8-*RS1* was associated with ocular inflammation as well as systemic antibodies against AAV that both increased in a dose-related fashion.^89^ Two clinical trials employing IVI of AAV2-based vectors to address LHON caused by mutations in mitochondrial *ND4* (ClinicalTrials.gov: NCT02161380 and NCT02064569) documented high antibody titers and anterior uveitis in some treated patients.^125^^,^^126^^,^^132^^,^^133^ Similarly, some patients who received high-dose IVI of AAV2 driving expression of a soluble VEGF neutralizing protein (AAV2-sFLT01) (2 × 10^10^ vg) for wet AMD (ClinicalTrials.gov: NCT01024998) developed pyrexia and intraocular inflammation. Similar inflammatory responses have been observed in NHP safety studies.^134^

Adverum Biotechnologies has an anti-VEGF product AAV.7m8 capsid carrying the gene for aflibercept in clinical testing that is being delivered by IVI. The first cohort (6 × 10^11^ vg) showed encouraging efficacy with 6/6 patients not requiring rescue injections of aflibercept post-treatment, maintenance of BCVA, maintenance or reduction in central retinal thickness, and durability out to 15 months. However, this cohort experienced recurrent uveitis that required additional steroid treatment (topical drops),^135^ resulting in a clinical hold pending review of the CMC.^136^ Upon resumption of the trial, subsequent cohorts were treated with a lower dose of vector (2 × 10^11^ vg), and changes to the prophylaxis steroid regimen were implemented, including a pretreatment oral versus topical steroid treatment regimen between two same-dose-level cohorts. The lower dose did appear to be associated with less post-treatment inflammation but was also less efficacious with 5/15 patients requiring rescue injections. Of interest is the observation that vector-induced uveitis appeared responsive to topical steroid drops, and in fact, the cohort treated with empiric difluprednate fared better with less inflammation post-treatment than did the same dose-level cohort treated with empiric oral prednisone.

Recent clinical data suggest that humoral immunity can play a role in limiting transduction for AAV delivered by IVI, although this is not believed to be the case with SRI. Results from the IVI AAV2-sFLT01 to treat wet AMD (ClinicalTrials.gov: NCT01024998) revealed a strong negative correlation with neutralizing antibodies (NAbs) to AAV2 and levels of sFLT01.^88^ This was the first clinical study to highlight the potential role of serum NAbs in limiting AAV-mediated transgene expression in the eye. In a separate study, AAV-mediated transgene expression was reported to be inversely correlated with the presence of NAbs in both serum and vitreous of macaque, with the latter showing a stronger correlation.^120^ As anti-VEGF AAV therapies pivot to clinical trials for progressive diabetic retinopathy (PDR) and diabetic macular edema (DME), both disorders, characterized by disruptions in the retinal blood barrier and concerns that NAbs will limit therapy, are heightened, given that the majority of the population is seropositive for AAV. For these reasons, there are efforts underway to develop AAV capsids that “escape” neutralization, thereby increasing the number of patients who would be amenable to IVI-delivered gene therapies.^112^ Whether sufficient transduction of the outer retina to effectively treat PR-mediated disease can be achieved by IVI AAV in the absence of neutralization or inflammation remains to be seen and will likely depend on the development of novel AAV capsids and/or immunosuppressive regimens.

As retinal gene therapy has evolved, we have come to understand that a “one-size-fits-all” approach will not work. Just as with other gene-therapy targets (e.g., hemophilia), we are learning that translating successful proof of concept studies in animal models will require more investigation aimed at identifying and overcoming hurdles that are intrinsic to the structure and physiology of the human eye and in some cases, the specific disease state of the target patient population. Fortunately, we have at our disposal a constantly improving set of tools (e.g., AAV capsids), delivery approaches, and diagnostic technologies that are likely to enable this endeavor. Taken together with its relative immune privilege and the much lower doses of vector needed to target the eye relative to other organs, the ocular gene-therapy field is likely to witness many additional successes.

### Hemophilia Gene Therapy: Moving beyond Proof of Concept

Success in hemophilia gene transfer has demonstrably arrived. As outlined in Tables 2 and 3, there are 20 clinical trials approaching, including a handful of pivotal trials and the first pending licensing application (Harrington et al., 2020, WFH Virtual Summit, conference).^137^, ^138^, ^139^, ^140^, ^141^, ^142^, ^143^, ^144^, ^145^, ^146^, ^147^, ^148^, ^149^, ^150^, ^151^, ^152^, ^153^^,^^154^, ^155^, ^156^ The field has largely converged on the use of systemically administered AAV vectors for hepatocyte expression of coagulation factors VIII (FVIII) and IX (FIX).^144^ Although AAV efforts predominate, lentiviral vectors for either systemic infusion^157^^,^^158^ or *ex vivo* transduction of hematopoietic stem cells^159^, ^160^, ^161^ or induced pluripotent stem cells^162^ are being pursued preclinically or as a phase I clinical trial.^156^ Although AAV clinical trial efforts have demonstrated repeated proof-of-concept successes, outstanding questions remain that are essential to address to realize hemophilia gene therapy’s potential. This point was recently highlighted by the FDA’s denial of the first biologics licensing application for a hemophilia gene-therapy product submitted by BioMarin Pharmaceutical for a hemophilia A (HA) vector; whereas the outcome surprised many, the FDA’s requested additional data aim to address unexplained observations thus far specific to its trial (e.g., questions around durability of expression^163^^,^^164^) that are undoubtedly important for all hemophilia gene-therapy efforts. More broadly, many of the lessons learned in hemophilia are applicable all systemic AAV gene-therapy efforts.

**Table 2:** Hemophilia B Gene Transfer Efforts in the Past Decade

Sponsor	Vector	Manufacturing Platform	Gene	Capsid	Dose (vg/kg)	OSA Approx. Mean FIX:C	Follow-up (Years)/Durability	ABR	Phase	Status
St. Jude/UCL	AAV8-FIX	mammalian	FIX	AAV8	2 × 10^12^	5%	8 years/stable	2	I/II	closed
Shire	BAX335	−	FIX-R338L	AAV8	3 × 10^12^	transient, except 1 subject 25%	N/A	N/A	I/II	closed
uniQure	AMT-060	insect	FIX	AAV5	2 × 10^13^	7%	4 years/stable	0.9	I/II	closed^a^
Ultragenyx	DTX101	−	FIX	rh-10	1.6^−5^ × 10^12^ to 5 × 10^12^	0%−1%	N/A	N/A	I/II	closed
Sangamo	SB-FIX	insect	FIX and ZFN	AAV6	−	−	−	−	−	I/II, recruiting
Freeline	FLT-180a	mammalian	FIX-R338L	AAVS3	4.5 × 10^11^	~40%	−	N/A	I/II	I/II, recruiting
					7.5 × 10^11^	2%−60%				
					9.75 × 10^11^	~50% to ~140%				
					1.5 × 10^12^	90% to ~250%				
Spark/Pfizer	SPK-9001	mammalian	FIX-R338L	SPK100	5 × 10^11^	40%	3 years/stable	0.4	I−III	phase III
uniQure	AMT-061	insect	FIX-R338L	AAV5	2 × 10^13^	40%	N/A	N/A	I−III	phase III^a^

vg/kg, vector genomes per kilogram; OSA, one-stage assay; Approx., approximate; FIX:C, factor IX activity; ABR, annual bleed rate; N/A, not applicable due to either subtherapeutic expression, or reported duration of follow-up is <1 year; FIX-R338L, factor IX-Padua; ZFN, zinc finger nuclease; −, data are not available; UCL, University College London.

a AMT-061 is AMT-060 containing the FIX-R338L mutation.

**Table 3:** Current Hemophilia A Gene Therapy Clinical Trials

Sponsor	Vector	Capsid	Manufacturing Platform	Dose (vg/kg)	Approx. Mean FVIII:C (OSA/CSA)	ABR	Phase	Follow-up (Years)/Durability	Status
BioMarin^149^, ^150^, ^151^	BMN- 270	AAV5	insect	4 × 10^13^	−/6%−20%^a^	0.5	I−III	3 years/declining expression	BLA application submitted, PDUFA action date 8/2020
				6 × 10^13^	−/4%−100%^b^	1.3		4 years/declining expression	
Spark/Roche	SPK-8016	−	−	−	−	−	I/II	−	recruiting phase I/II
Spark/Roche^152^	SPK-8011	LK03	mammalian	5 × 10^11^	8%−12%/−	0.2	I/II	2.5−3.3 years/stable expression	recruiting phase I/II
				1 × 10^12^	3%−22%/−	1.7	I/II	2−2.5 years/stable expression	
				2 × 10^12^	0%−25%/−	1.7^c^	I/II	1−1.5 years/unknown	
Sangamo/Pfizer (Harrington et al., 2020, WFH Virtual Summit, conference)^153^	SB-525	AAV2/6	insect	9 × 10^11^	−	−	I/II	0.6−1.2 years/unknown	phase III planned
				2 × 10^12^	−	−			
				1 × 10^13^	−	−			
				3 × 10^13^	30%−250%/20%−160%	N/A			
UCL^154^	GO-8	AAV8	mammalian	6 × 10^11^	8%−64%/−	N/A	I/II	≤1 year	recruiting phase I/II
				2 × 10^12^					
Ultragenyx/Bayer^155^	DTX-201	hu37	mammalian	5 × 10^12^	5%−20%/−	N/A	I/II	≤1 year	recruiting phase I/II
				1 × 10^13^	8%−40%/−				
				2 × 10^13^	−				
Takeda	TAK-754	AAV8	mammalian	−	−	−	I/II	−	active, not recruiting
Medical College of Wisconsin^156^	CD34^+^ PBSC, lentiviral-FVIII	not AAV	−	−	−	−	I	−	recruiting

N/A, not applicable, or follow-up duration is <1 year; FVIII:C, factor VIII activity; CSA, chromogenic assay; BLA, Biologics License Application; PDUFA, Prescription Drug User Fee Act.

a Reported FVIII activity at 2-year follow-up.

b Reported FVIII activity at 3-year follow-up.

c Evaluation of bleeding excluded 2 of 7 total subjects who lost transgene expression.

HA and hemophilia B (HB) are X-linked monogenic disorders resulting from decreased/absent function of coagulation FVIII or FIX.^165^^,^^166^ Whereas FIX is endogenously synthesized in hepatocytes, FVIII is synthesized predominantly in liver sinusoidal endothelial cells, such that FVIII is ectopically expressed in clinical trials.^167^, ^168^, ^169^, ^170^ Hemophilia patients experience recurrent, spontaneous, or trauma-induced bleeding classically into joints, resulting in disabling arthropathy. Bleeding sequelae correlate with plasma FVIII/FIX activity (FVIII:C/FIX:C), such that spontaneous hemorrhage occurs in severe HA/HB (FVIII/FIX:C <1% of normal), less commonly in moderate HA/HB (FVIII/FIX:C 1%–5% of normal), and typically only following trauma in mild HA/HB (FVIII/FIX:C >5%–40% of normal).^171^ Thus, a modest amount of FVIII/FIX expression has a large clinical benefit. Hemophilia is treated with intravenous enzyme replacement therapy (ERT) delivered prophylactically to prevent or on demand for bleeding.^172^^,^^173^ Although effective, ERT is limited by an approximately 40% noncompliance rate, costs ≥$200,000 annually, and advances arthropathy.^172^, ^173^, ^174^, ^175^

#### Systemic AAV Safety Considerations

There have been minimal short-term safety concerns from systemic AAV administration at the 120-fold range of vector doses (5 × 10^12^ to 6 × 10^13^ vg/kg) employed in hemophilia trials (Tables 1 and 2). Approximately 5 subjects across 3 trials experienced self-limited, vector-infusion reactions at doses of 2 × 10^12^−6 × 10^13^ vg/kg, characterized by fever, myalgias, and/or hypotension, which appear consistent with an innate immune response.^141^^,^^150^^,^^152^ Hints of dose-limiting toxicities of systemic AAV infusion are supported by observations in nonhemophilia clinical trials that include: approval of Zolgensma (1 × 10^14^ vg/kg) with a boxed safety warning of hepatotoxicity postinfusion, suspected complement activation in DMD trials (5 × 10^13^−3 × 10^14^ vg/kg) with resultant cytopenias and renal toxicity, and 3 deaths in the 3 × 10^14^-vg/kg cohort of an XLMTM trial in the setting of progression of pre-existing hepatobiliary disease, 4−6 weeks after vector infusion.^13^^,^^176^, ^177^ Composite analysis of these observations is ongoing and relevant for all systemic AAV work and includes potential inherent differences between AAV serotypes with respect to immune responses.

Given that all AAV serotypes transduce hepatocytes, long-term safety considerations have predominantly focused on hepatotoxicity risk, including risk of insertional mutagenesis resulting in hepatocellular carcinoma (HCC). Whereas predominantly nonintegrating, preclinical data suggest AAV integration events occur in a dose-dependent manner with a preference for integration at sites of active transcription, tissue-specific promoters are protective against oncogenesis.^178^, ^179^, ^180^ Collectively, this would support use of the lowest possible effective vector dose with a hepatocyte-specific promoter to minimize genotoxicity risk. Hemophilia is a provocative model to study AAV-related HCC genotoxicity because ∼90% of severe hemophilia patients >35 years contracted iatrogenic hepatitis C virus (HCV), an established risk factor for developing HCC.^181^, ^182^, ^183^, ^184^, ^185^ Recently published 15-year follow-up data of 4 HB subjects supported no evidence of hepatotoxicity or other long-term toxicities; however, conclusions were limited by the lack of confirmed transgene persistence in transduced hepatocytes.^186^

#### FVIII/FIX Transgene Safety: Thrombosis and Allo-inhibitory Antibody Formation Risk

All HA clinical trials use a B-domain-deleted FVIII (BDD-FVIII) transgene, which retains full procoagulant function while meeting AAV packaging constraints (∼4.7 kb) and enhancing expression.^187^, ^188^, ^189^ Nearly all HA trials use a standard FVIII B-domain heavy and light chain linker, FVIII-SQ.^190^ A single trial is using an alternative BDD-FVIII, FVIII-V3, wherein 6 additional N-linked glycosylation sites were added to the SQ linker to improve expression.^154^^,^^191^ HB trials have universally adapted the FIX-Padua (FIX-R338L) transgene, which affords ∼8-fold greater specific activity relative to wild-type FIX.^192^^,^^193^

Given the known incidences of allo-inhibitory antibodies, “inhibitors,” formation in severe HA (30%) and HB (5%) and the use of FVIII-SQ and FIX-R338L variant transgenes raise the theoretical concern of an immune response to the transgene-derived protein.^194^^,^^195^ Consistent with 2 decades of experience with rFVIII-SQ ERT, demonstrating no increased inhibitor risk, no HA gene-therapy subjects have developed an inhibitor.^196^, ^197^, ^198^ Further, the success and safety of FIX-R338L for HB gene-therapy efforts support gain-of-function FVIII variants (reviewed in Samelson-Jones and Arruda^199^) and may similarly be safe with enhanced therapeutic benefit. However, trial enrollment criteria require significant prior FVIII/FIX protein exposure and exclude patients with a history of inhibitor. As such, available gene-therapy data reflect patients least likely to develop an inhibitor. Nonetheless, low risk of inhibitor formation postgene therapy is further supported by mouse and canine data demonstrating the ability of hepatocyte-directed gene transfer to induce FVIII, FIX, or FIX-R338L tolerance.^200^, ^201^, ^202^, ^203^ These preclinical successes support future investigation of gene transfer for tolerance induction, which is particularly relevant for HA patients with inhibitors^204^ and other disorders (e.g., Pompe disease; reviewed in Doerfler et al.^205^).

With respect to prothrombotic risk, available biochemical data suggest FIX-R338L is activated and inactivated analogous to wild-type FIX and not inherently prothrombotic.^193^ Further, mouse data demonstrated thrombotic events in FIX-R338L and wild-type FIX correlated with supratherapeutic FIX:C and were not specific to FIX-R338L.^201^ Epidemiological studies have outlined that supraphysiologic FIX:C is a modest independent risk-factor venous thrombosis (odds ratio [OR] 1.8−4.0) relative to supraphysiologic FVIII (OR 8.8−21.3).^206^, ^207^, ^208^ Multiple FIX-R338L trials achieved FIX:C in the range of normal or mild hemophilia without safety concerns. In recent data, a single FIX-R338L trial reported thrombosis in a subject who achieved FIX:C > 200% of normal (by one-stage assay [OSA]) with potentially multiple contributing prothrombotic comorbidities, including, obesity, kidney failure, and recent cessation of a direct-Xa oral anticoagulant^145^ (a suspected rebound prothrombotic state^209^). Nonetheless, this observation, paired with known epidemiological data of factor activity and thrombosis, underscores the importance of maintaining expression within a therapeutic window, particularly in the setting of prothrombotic comorbidities.

#### HA and HB Gene-Therapy Efficacy Questions

Epidemiological data may guide target therapeutic FVIII/FIX expression. Potentially targeting FVIII/FIX:C, >10%–150% of normal postgene transfer is supported by both the aforementioned prothrombotic risks associated with supraphysiologic FVIII/FIX:C^206^, ^207^, ^208^ and available HA natural history data that demonstrate that FVIII:C of ≥12% of normal (by OSA) is adequate to prevent spontaneous joint bleeding.^210^ Importantly, both transgene products, FVIII-SQ and FIX-R338L, demonstrate variable activity measurements in OSA versus chromogenic assay (CSA) determinants of factor activity that are reproducible across trials.^144^^,^^145^^,^^149^^,^^152^ Unlike FVIII-SQ, FIX-R338L differences are not gene-therapy specific, suggesting the assay discrepancies are related to FIX-R338L biochemistry.^192^^,^^201^^,^^211^^,^^212^ The understanding of which assay best predicts *in vivo* hemostatic function of hepatocyte-derived FVIII/FIX-R338L is relevant to guide clinical management because sustained FVIII:C/FIX:C, depending on the assay used, crosses clinical thresholds of mild/moderate hemophilia,^152^^,^^155^ normal FVIII:C/FIX:C (Harrington et al., 2020, WFH Virtual Summit, conference),^145^^,^^146^ and supratherapeutic FVIII:C/FIX:C (Harrington et al., 2020, WFH Virtual Summit, conference).^145^ Preliminary FVIII antigen data correlate with CSA-determined FVIII:C,^153^ whereas preliminary phenotypic data supporting OSA-determined FVIII:C correlate with hepatocyte-derived FVIII *in vivo* function,^152^ suggesting that hepatocyte-derived FVIII may be more easily converted from its procofactor to cofactor state.

Accurate measurement of transgene hemostatic function is necessary to outline a tolerated range of therapeutic FVIII/FIX expression. Consistent with reports in a chimeric mouse model of human hepatocytes demonstrating an up to 7-fold difference in transduction between mice,^213^ multiple HA and HB trials have demonstrated ≥10-fold variability in FVIII:C/FIX:C with the same vector and dose (Harrington et al., 2020, WFH Virtual Summit, conference).^145^^,^^149^^,^^150^^,^^153^ Part of this variability may be explained, in part, by a hypothesized cellular immune response to capsid peptides presented by transduced hepatocytes that can result in reduced or loss of transgene expression, thereby limiting efficacy.^214^ Additionally, vector CpG motifs may potentially be immune stimulatory and contribute to a capsid response;^215^ indeed, cassette CpG enrichment has been implicated in loss of transgene expression despite steroid intervention in 3 HB trials.^140^^,^^143^^,^^216^ More broadly, the multiple steps from vector infusion to transgene expression (reviewed in Li and Samulski^217^) with accompanying potential for variability likely means it will be necessary to tolerate a range of therapeutic transgene expression, albeit hopefully less than 10-fold.

Lastly, whereas undefined, durability of transgene expression in HA/HB clinical trials may inform other AAV hepatocyte-directed gene therapies. Clinical data from the first successful systemic AAV trial, conducted in HB,^163^^,^^164^ mirror observations in canine HA and HB data after AAV-mediated gene transfer, demonstrating durable expression 8 years postgene transfer.^139^^,^^164^^,^^218^^,^^219^ In contrast, the first successful HA gene-therapy trial reported declining expression at years 1−4 postvector,^151^ such that published 3-year FVIII:C declined by ∼35%–80% of year 1 values in 6 of 7 subjects in the 6 × 10^13^-vg/kg cohort, the anticipated licensed dose. Recently, another HA trial demonstrated stable and durable FVIII expression for 2−3.3 years in 5 subjects (5 × 10^11^−1 × 10^12^ vg/kg cohorts), supporting the potential for stable and durable hepatocyte-derived FVIII expression.^152^ Several hypotheses have been generated to potentially explain the decline in FVIII expression observed in the first HA trial^150^ that include: a FVIII unfolded protein response (UPR) reported in heterologous expression systems with contrasting data in HA mouse models post-AAV-mediated FVIII gene transfer^220^, ^221^, ^222^ or vector-specific properties (e.g., manufacturing platform, cassette size, dose, capsid, etc.). Notably, in the first successful HA trial, the single subject with sustained expression at year 3 had FVIII:C ∼100% of normal, whereas FVIII:C among the subjects that lost expression began or crossed into the range mild and moderate HA,^150^^,^^151^ values that were stable and durable in another trial.^152^ Although preliminary and not definitive, these data would suggest that observed decline in FVIII:C or stable expression is not FVIII expression dependent, making a FVIII UPR an unlikely predominant culprit for loss of expression. Success in HA gene therapy is recent, and ongoing observation will inform expectations of FVIII durability.

Durability of expression is particularly relevant when considering that all subjects universally develop high-titer AAV NAbs following systemic AAV vector infusion, which likely preclude repeat administration of the same AAV serotype. Whereas the majority of data supporting high-titer NAb preclude systemic AAV efficacy, a sponsor has reported detectable transgene expression following systemic AAV5 vector in NHPs or humans who are AAV5 NAb positive.^148^^,^^223^ Two ongoing trials, which may provide additional insight, enroll Nab-positive subjects (ClinicalTrials.gov: NCT03520712 and NCT03569891). Nonetheless, the longevity of the AAV humoral immune is supported by a 15-year follow-up of the first systemic AAV trial, demonstrating persistent, multi-serotype, cross-reactive AAV NAbs to the infused vector serotype and all others tested.^186^ These data are consistent with observations in humans following AAV environmental exposure and systemic AAV vector infusion in animal models that demonstrated persistent, multi-serotype, cross-reactive NAbs.^224^, ^225^, ^226^, ^227^, ^228^ Collectively, available data suggest that with current methods, a once-in-a-lifetime AAV vector infusion is both the goal of therapy and compulsory due to NAb formation.

In summary, three decades of investigation have refined AAV gene therapy for hemophilia to achieve repeated proof-of-concept success and outlined outstanding questions needed to move gene therapy forward. Exceptional activity in hemophilia gene therapy imparts considerable optimism that answers will be identified to progress efforts closer to the ultimate goal of safely and reliably achieving durable and stable FVIII/FIX therapeutic expression able to ameliorate hemophilia phenotype.

### LSDs: Clinical Gene-Therapy Overview

LSDs are a group of inherited metabolic disorders that are characterized by the accumulation of macromolecules inside lysosomes. Collectively, LSDs are relatively common disorders (1:5,000 to 1:5,500), although individually rare.^229^, ^230^, ^231^ LSDs affect multiple organ systems but have one defining system predominating, such as hypertrophic cardiomyopathy in patients with infantile Pompe disease, CNS deficits in children with CNL2, or hepatosplenomegaly in children with Gaucher disease type I.^230^ Given their clinical heterogeneity, finding effective treatments has been a challenge with the only approved therapy, ERT, creating secondary complications from increased survival.^232^ As the prevalence of these disorders increases with the advent of newborn screening, new therapeutic interventions, such as gene replacement, will become critical in addressing all manifestations of LSDs.^230^^,^^233^^,^^234^

In this section, we discuss the progress that has been made in developing gene replacement therapies for some of the more common LSDs: Pompe disease, Gaucher disease, Fabry disease, mucopolysaccharidoses (MPS) disease, and neuronal ceroid lipofuscinoses (NCLs) (Table 1).

#### Pompe Disease

Pompe disease is a glycogen storage disorder that leads to glycogen accumulation in muscle and motoneurons.^235^^,^^236^ Lack of acid alpha-glucosidase (GAA) activity in patients with severe/early onset results in a clinical pathology that includes profound weakness and hypotonia and cardiorespiratory failure.^237^ Recently, newborn screening efforts have discovered a significantly higher incidence of up to 1:9,500, making gene therapy a rational solution for presymptomatic treatment. For Pompe disease, four phase I/II gene-therapy studies have achieved approval from the FDA. Corti et al.^238^ and Byrne et al.^239^ conducted a first-in-human trial injecting AAV1-cytomegalovirus (CMV)-GAA into the diaphragms of nine patients with ventilatory insufficiency in an effort to correct the respiratory dysfunction characteristic of early-onset Pompe disease (EOPD). The results indicated that the vector was both safe to use and effective at improving ventilatory performance in all subjects.^238^^,^^240^^,^^241^

Another clinical trial from the University of Florida is currently being conducted in patients with late-onset Pompe disease (LOPD). The study evaluates the ability to repeat the administered of AAV (rAAV9-DES-hGAA) after a period of time to maintain therapeutic levels of GAA in adult Pompe patients^242^ (ClinicalTrials.gov: NCT02240407). Preliminary results suggest that pharmacological modulation of the immune system using rituximab and sirolimus, before and at the time of AAV dosing, prevents the formation of antibody and allows for repeated AAV dosing. With early intervention due to newborn screening, this is an important observation for LSDs affecting the muscle and the liver, where somatic growth may cause a decline in genome copy number. The University of Florida team, in collaboration with an NIH Clinical Center team led by Dr. Bönneman, is also conducting a study to evaluate the safety and efficacy of an intravenous dose of AAV-GAA in children with EOPD. Patients enrolled in this study will receive the same immunomodulation regimen used in the LOPD study to manage immune responses to AAV and GAA.

In addition to the work conducted at the University of Florida, a few other programs have initiated clinical programs to test their own AAV vectors in patients with Pompe disease.

Spark Therapeutics has been approved to enroll in phase I/II vector-mediated liver gene transfer for GAA in LOPD. This approach relies on stable expression of GAA in the liver achieved via AAV vector-mediated gene transfer, resulting in cross-correction in peripheral organs with no evident immunogenicity against the transgene (ClinicalTrials.gov: NCT04093349). The preclinical data revealed promising results, suggesting sustained plasma levels of GAA.^243^ 10 months postdosing, all GAA knockout (Gaa−/−) mice showed decreased glycogen accumulation; increased survival; and improved cardiac, respiratory, and muscle function compared to wild-type mice. In comparison to ERT, the current standard of care for Pompe disease, SPK-3006 was also more effective at breaking down and clearing excess glycogen build-up in refractory muscle groups typically mute to the effects of ERT (Mendoza, 2018, International Congress of the World Muscle Society, conference).^243^ First-in-human clinical studies are currently under way at the University of California Irvine Health (ClinicalTrials.gov: NCT04093349).

Asklepios Biopharmaceutical has also been approved to enroll in a phase I/II open-label trial to assess the safety and determine the bioactivity of ACTUS-101 (AAV2/8LSPhGAA) at two dose levels in human subjects with LOPD. With the use of a liver-specific promoter, this AAV vector was manufactured to express GAA specifically in the liver accompanied by GAA secretion and receptor-mediated uptake of GAA in the cardiac and skeletal muscle. The central hypothesis is that continuous GAA production from a liver depot will provide more benefit than ERT in Pompe disease (ClinicalTrials.gov: NCT03533673). On January 22, 2019, the first patient in the phase I/II clinical study was dosed with ACTUS-101.^244^

#### Gaucher Disease

Gaucher disease, the most common of the LSDs, is an autosomal recessive LSD caused by a deficiency in lysosomal enzyme acid beta-glucosidase (glucocerebrosidase). Although heterogenous in its presentation based on type, the main clinical effects are hepatosplenomegaly, neurodegeneration, bone disease, and pulmonary complications.^245^ There have been two *ex vivo* gene replacement trials to date for Gaucher disease. With the use of a retroviral transduction of peripheral blood or CD34^+^ cells, Dunbar and Kohn^246^ examined the safety of a G1Gc vector that uses the viral long terminal repeat (LTR) promoter to express the human glucocerebrosidase cDNA.^247^ The study did result in transient low-level expression of corrected cells, however too low to result in any clinical benefit or increased glucocerebrosidase enzyme activity.^248^ In the second, currently active gene-therapy trial, AVROBIO is using an *ex vivo* lentiviral-based gene-therapy approach designed to result in a stable integration of the desired genes into patient-derived hematopoietic stem cells (ClinicalTrials.gov: NCT04145037). Preclinical trials showed positive results in lentiviral vector integration into parent stem cells and effective replication of integrated progeny cells.^249^

#### Fabry Disease

Fabry disease is characterized by deficient activity of α-galactosidase A (α-Gal A), resulting in accumulation of glycolipids (globotriaoslyceramide [Gb3] and globotriaosylsphingosine [LysoGb3]) in various tissues. Clinical manifestation includes progressive kidney failure, heart disease, cerebrovascular disease, skin lesions, and other abnormalities.^250^^,^^251^ Although ERT is the current standard of care for patients with Fabry, it does have its limitations producing increased interest in gene replacement therapies.^252^^,^^253^ Of the five gene replacement studies to date, two of them use an *ex vivo* approach (AVROBIO and University Health Network, Toronto), whereas two others use an *in vivo* approach (Sangamo Therapeutics and Freeline Therapeutics).

AVROBIO’s vector (AVR-RD-01) is derived from hemopoietic stem cells to which the gene encoding AGA is added in an *ex vivo* process using a lentiviral vector. In the ongoing phase I/II clinical trial (ClinicalTrials.gov: NCT03454893), interim data showed all four patients dosed in the phase I portion displayed increased AGA enzyme activity level above the levels of patients with classical Fabry (ESGCT Annual Meeting abstract, Lausanne, 2018).^251^^,^^254^ Preclinical studies for the University Health Network’s lentiviral product also showed promising results.^255^ University Health Network’s lentiviral α-Gal A transduced stem cell therapy is currently being tested at several Canadian clinical trial sites (ClinicalTrials.gov: NCT02800070) (ASGCT Annual Meeting abstract, San Diego, 2010).

Another first-in-human phase I/II trial sponsored by Sangamo Therapeutics is using an AAV vector (rAAV2/6) to produce the deficient enzyme at clinically significant levels. ST-920 is an AAV vector encoding the cDNA for human α-Gal A with a liver-specific promoter designed to enable a patient’s liver to produce a continuous supply of the α-Gal A enzyme (ClinicalTrials.gov: NCT04046224). The constant production is anticipated to reduce Gb3 and LysoGb3.^251^^,^^256^

Similarly, Freeline Therapeutics is using an AAV vector (rAAV8) with a liver-specific promoter to produce sustained high levels of α-Gal A. Preliminary data on the starting dose in the dose-escalation study reported a 3- to 4-fold increase in plasma α-Gal A activity by week 4 postdose and was sustained through the data cutoff.^251^ On March 10, 2020, the European Commission granted orphan drug designation for FLT190 for the treatment of Fabry disease, based on a positive opinion from the Committee for Orphan Medicinal Products of the European Medicines Agency.^257^ 4D Molecular Therapeutics is also pursuing a gene-therapy study that is recently open for enrollment.

#### MPS Type III (MPS III)—Sanfilippo Syndrome

MPS III, also known as Sanfilippo syndrome, is a progressive disorder characterized by the accumulation of glycosaminoglycan in neural cells.^254^ MPS III primarily affects the CNS, resulting in neurodegeneration, progressive intellectual disability, and developmental regression. As the brain is the most affected organ with MPS III, brain-targeted gene replacement therapies have become increasingly studied for both MPS IIIA and MPS IIIB by different sponsors using similar approaches.

LYSOGENE, a biotechnology company from France, used AAVrh.10 to carry the human SGSH and SUMF1 cDNAs for the treatment of MPS IIIA. The therapeutic vector AAVrh.10-hMPS3A was administered to four children in a phase I first-in-human trial via intracerebral injections. All four patients were followed for a year postdose. The results of the trial proved the method of administration to be safe for direct AAV vector delivery into the CNS. Neurocognitive evaluations suggested a cognitive benefit in the youngest child, whereas a more limited benefit in the three older patients.^254^^,^^258^

uniQure Biopharma B.V., also using intracranial injections, administered an rAAV2/5 vector encoding human α-N-acetylglucosaminidase (NAGLU) in seven children for the treatment of MPS IIIB. Following administration, NAGLU activity in the CNS was found to be increased from baseline with sustained enzyme production by brain cells. All patients displayed improvements on their neurocognitive evaluations with the youngest functioning close to that of a healthy child. These results suggest that this approach could prevent or slow cognitive decline in children with MPS IIIB.^259^

Additional *in vivo* and *ex vivo* phase I/II gene-therapy clinical trials for MPS are listed in Table 1.

#### NCLs

NCLs are a group of inherited, autosomal, progressive childhood neurodegenerative disorders characterized clinically by dementia, epilepsy, and vision loss through retinal degeneration. NCLs are caused by an accumulation of ceroid lipofuscin in the neuronal cells in the brain and in the retina. To date, there are 13 forms of NCLs, each having distinct defects in genes encoding proteins in the lysosomal system.^260^, ^261^, ^262^ Currently, there are no treatments approved for NCLs. In an effort to find an efficacious therapy for NCL, novel gene replacement strategies have been explored.

Worgall et al.^263^ developed an AAV serotype 2 vector expressing the human CLN2 cDNA (AAV2_CU_hCLN2) and administered the vector in the CNS of 10 children with late infantile NCL (LINCL).^262^ In comparison to the control subjects, disease progression assessed by CNS imaging was slower, although not statistically significant, showing reduced gray matter and ventricular volume. Notably, results from the modified Hamburg scale postdose demonstrated significantly slower functional decline compared to the control group.^262^^,^^263^

Also with the use of an *in vivo* approach, Cain et al. ^264^ developed a scAAV9 vector expressing the hCLN6 gene under the control of a CB hybrid promoter.^262^ scAAV9.CB.hCLN6 was injected intrathecally into the CSF of 4-year-old NHPs and intracerebroventricularly (i.c.v.) into mice. High transgene expression was found throughout the brain and spinal cord of the NHPs with very few lab abnormalities. The i.c.v. injection administered to the mice also exhibited promising results, including prevention of classic CLN6 brain disease pathology, correction of behavioral deficits, and increased survival. Taken together, the results indicate the efficacy and safety of scAAV9.CB.hCLN6.^262^^,^^264^ This approach was adopted by Amicus Therapeutics and is currently being studied clinically in patients with Batten disease (ClinicalTrials.gov: NCT02725580). Additional *in vivo* phase I/II gene-therapy clinical trials for NCLs are listed in Table 4.

**Table 4:** Summary of Gene Replacement Clinical Trials for Select LSDs

Disease	Product	Sponsor	NCT (ClinicalTrials.gov:)	Route
Pompe disease	rAAV1-CMV-hGAA	University of Florida, USA	NCT00976352	i.m.
	rAAV9-DES-hGAA	University of Florida, USA	NCT02240407	i.m.
	SPK-3006	Spark Therapeutics	NCT04093349	i.v.
	ACTUS-101 (AAV2/8LSPhGAA)	Asklepios Biopharmaceutical	NCT03533673	i.v.
Gaucher disease	G1Gc vector	National Institute of Neurological Disorders and Stroke (NINDS)	NCT00001234	i.v.
	mAVR-RD-02	AVROBIO	NCT04145037	i.v.
Fabry disease	RV-*GLA*	NINDS	NCT00001234	i.v.
	mAVR-RD-01	AVROBIO	NCT03454893	i.v.
	FLT190	Freeline Therapeutics	NCT04040049	i.v.
	ST-920 (rAAV2/6)	Sangamo Therapeutics	NCT04046224	i.v.
	lentivirus α-Gal A transduced stem cells	University Health Network, Toronto, ON, Canada	NCT02800070	i.v.
Mucopolysaccharidoses (MPS)−all types	RV-*IDS*	Eunice Kennedy Shriver National Institute of Child Health and Human Development (NICHD)	NCT00004454	i.v.
	SAF-301 (AAVrh.10-*SGHS* and *SUMF*)	LYSOGENE	NCT01474343	i.c.
	LYS-SAF302 (AAVrh.10-*SGHS*)	LYSOGENE	NCT03612869	i.c.
	rAAV2/5-hNAGLU	uniQure Biopharma B.V.	NCT03300453	i.c.
	lentivirus transduced stem cells	University of Manchester	NCT04201405	i.v.
	RGX-111 (AAV9, α-L-iduronidase cassette)	REGENXBIO	NCT03580083	i.c.
	RGX-121 (AAV9, iduronate-2-sulfatase cassette)	REGENXBIO	NCT03566043	i.c.
	AAV2/8.TBG.hARSB	Fondazione Telethon	NCT03173521	i.v.
	rAAV9.CMV.hNAGLU	Abeona Therapeutics	NCT03315182	i.v.
	ABO-102 (scAAV9.U1a.hSGSH)	Abeona Therapeutics	NCT02716246, NCT01474343	i.v.
	lentivirus transduced stem cells	IRCCS San Raffaele	NCT03488394	i.v.
Neuronal ceroid lipofuscinoses, late infantile (CLN2)	AAVrh.10-*CLN2*	Weill Medical College of Cornell University	NCT01161576	i.c.
	AAV2CUhCLN2	Weill Medical College of Cornell University	NCT00151216	i.c.
	AAVrh.10CUCLN2	Weill Medical College of Cornell University	NCT01414985	i.c.
	AT-GTX-501 (scAAV9.CB.CLN6)	Amicus Therapeutics	NCT02725580	i.t.
	AT-GTX-502 (scAAV9.P546.CLN3)	Amicus Therapeutics	NCT03770572	i.t.

i.m., intramuscular; i.v., intravenous; i.c., intracerebral; i.t., intrathecal.

### Conclusions

The advent of advanced therapeutics in lysosomal storage disease provides for new opportunities for patients and hopefully transformative therapeutic opportunities. As the field matures, we hope that additional safe and effective therapeutic options will be widely available. Challenges still exist for establishing a network of qualified providers to administer gene-therapy medicinal products.

## References

[1] Burghes A.H., Beattie C.E. (2009). Spinal muscular atrophy: why do low levels of survival motor neuron protein make motor neurons sick?. Nat. Rev. Neurosci..

[2] Verhaart I.E.C., Robertson A., Wilson I.J., Aartsma-Rus A., Cameron S., Jones C.C., Cook S.F., Lochmüller H. (2017). Prevalence, incidence and carrier frequency of 5q-linked spinal muscular atrophy - a literature review. Orphanet J. Rare Dis..

[3] Sugarman E.A., Nagan N., Zhu H., Akmaev V.R., Zhou Z., Rohlfs E.M., Flynn K., Hendrickson B.C., Scholl T., Sirko-Osadsa D.A., Allitto B.A. (2012). Pan-ethnic carrier screening and prenatal diagnosis for spinal muscular atrophy: clinical laboratory analysis of >72,400 specimens. Eur. J. Hum. Genet..

[4] Lefebvre S., Bürglen L., Reboullet S., Clermont O., Burlet P., Viollet L., Benichou B., Cruaud C., Millasseau P., Zeviani M. (1995). Identification and characterization of a spinal muscular atrophy-determining gene. Cell.

[5] Monani U.R., Lorson C.L., Parsons D.W., Prior T.W., Androphy E.J., Burghes A.H., McPherson J.D. (1999). A single nucleotide difference that alters splicing patterns distinguishes the SMA gene SMN1 from the copy gene SMN2. Hum. Mol. Genet..

[6] Lorson C.L., Hahnen E., Androphy E.J., Wirth B. (1999). A single nucleotide in the SMN gene regulates splicing and is responsible for spinal muscular atrophy. Proc. Natl. Acad. Sci. USA.

[7] Feldkötter M., Schwarzer V., Wirth R., Wienker T.F., Wirth B. (2002). Quantitative analyses of SMN1 and SMN2 based on real-time lightCycler PCR: fast and highly reliable carrier testing and prediction of severity of spinal muscular atrophy. Am. J. Hum. Genet..

[8] Finkel R.S., McDermott M.P., Kaufmann P., Darras B.T., Chung W.K., Sproule D.M., Kang P.B., Foley A.R., Yang M.L., Martens W.B. (2014). Observational study of spinal muscular atrophy type I and implications for clinical trials. Neurology.

[9] Foust K.D., Nurre E., Montgomery C.L., Hernandez A., Chan C.M., Kaspar B.K. (2009). Intravascular AAV9 preferentially targets neonatal neurons and adult astrocytes. Nat. Biotechnol..

[10] Duque S., Joussemet B., Riviere C., Marais T., Dubreil L., Douar A.M., Fyfe J., Moullier P., Colle M.A., Barkats M. (2009). Intravenous administration of self-complementary AAV9 enables transgene delivery to adult motor neurons. Mol. Ther..

[11] Foust K.D., Wang X., McGovern V.L., Braun L., Bevan A.K., Haidet A.M., Le T.T., Morales P.R., Rich M.M., Burghes A.H., Kaspar B.K. (2010). Rescue of the spinal muscular atrophy phenotype in a mouse model by early postnatal delivery of SMN. Nat. Biotechnol..

[12] Dominguez E., Marais T., Chatauret N., Benkhelifa-Ziyyat S., Duque S., Ravassard P., Carcenac R., Astord S., Pereira de Moura A., Voit T., Barkats M. (2011). Intravenous scAAV9 delivery of a codon-optimized SMN1 sequence rescues SMA mice. Hum. Mol. Genet..

[13] Mendell J.R., Al-Zaidy S., Shell R., Arnold W.D., Rodino-Klapac L.R., Prior T.W., Lowes L., Alfano L., Berry K., Church K. (2017). Single-Dose Gene-Replacement Therapy for Spinal Muscular Atrophy. N. Engl. J. Med..

[14] Al-Zaidy S., Pickard A.S., Kotha K., Alfano L.N., Lowes L., Paul G., Church K., Lehman K., Sproule D.M., Dabbous O. (2019). Health outcomes in spinal muscular atrophy type 1 following AVXS-101 gene replacement therapy. Pediatr. Pulmonol..

[15] Lowes L.P., Alfano L.N., Arnold W.D., Shell R., Prior T.W., McColly M., Lehman K.J., Church K., Sproule D.M., Nagendran S. (2019). Impact of Age and Motor Function in a Phase 1/2A Study of Infants With SMA Type 1 Receiving Single-Dose Gene Replacement Therapy. Pediatr. Neurol..

[16] Al-Zaidy S.A., Kolb S.J., Lowes L., Alfano L.N., Shell R., Church K.R. (2019). AVXS-101 (Onasemnogene Abeparvovec) for SMA1: Comparative Study with a Prospective Natural History Cohort. Neuromuscul. Dis..

[17] Waldrop M.A., Karingada C., Storey M.A., Powers B., Iammarino M.A., Miller N.F. (2020). Gene Therapy for Spinal Muscular Atrophy: Safety and Early Outcomes. Pediatrics.

[18] Hordeaux J., Buza E.L., Dyer C., Goode T., Mitchell T.W., Richman L., Denton N., Hinderer C., Katz N., Schmid R. (2020). Adeno-Associated Virus-Induced Dorsal Root Ganglion Pathology. Hum. Gene Ther..

[19] Koenig M., Hoffman E.P., Bertelson C.J., Monaco A.P., Feener C., Kunkel L.M. (1987). Complete cloning of the Duchenne muscular dystrophy (DMD) cDNA and preliminary genomic organization of the DMD gene in normal and affected individuals. Cell.

[20] Campbell K.P. (1995). Three muscular dystrophies: loss of cytoskeleton-extracellular matrix linkage. Cell.

[21] Brooke M.H., Griggs R.C., Mendell J.R., Fenichel G.M., Shumate J.B. (1981). The natural history of Duchenne muscular dystrophy: a caveat for therapeutic trials. Trans. Am. Neurol. Assoc..

[22] Mendell J.R., Shilling C., Leslie N.D., Flanigan K.M., al-Dahhak R., Gastier-Foster J., Kneile K., Dunn D.M., Duval B., Aoyagi A. (2012). Evidence-based path to newborn screening for Duchenne muscular dystrophy. Ann. Neurol..

[23] Al-Zaidy S., Rodino-Klapac L., Mendell J.R. (2014). Gene therapy for muscular dystrophy: moving the field forward. Pediatr. Neurol..

[24] Mendell J.R., Rodino-Klapac L.R., Sahenk Z., Roush K., Bird L., Lowes L.P., Alfano L., Gomez A.M., Lewis S., Kota J., Eteplirsen Study Group (2013). Eteplirsen for the treatment of Duchenne muscular dystrophy. Ann. Neurol..

[25] Charleston J.S., Schnell F.J., Dworzak J., Donoghue C., Lewis S., Chen L., Young G.D., Milici A.J., Voss J., DeAlwis U. (2018). Eteplirsen treatment for Duchenne muscular dystrophy: Exon skipping and dystrophin production. Neurology.

[26] England S.B., Nicholson L.V., Johnson M.A., Forrest S.M., Love D.R., Zubrzycka-Gaarn E.E., Bulman D.E., Harris J.B., Davies K.E. (1990). Very mild muscular dystrophy associated with the deletion of 46% of dystrophin. Nature.

[27] Mendell J.R., Sahenk Z., Lehman K., Nease C., Lowes L.P., Miller N.F., Iammarino M.S., Alfano L.N., Nicholl A., Al-Zaidy S. (2020). Assessment of Systemic Delivery of rAAVrh74.MHCK7.micro-dystrophin in Children With Duchenne Muscular Dystrophy: A Nonrandomized Controlled Trial. JAMA Neurol..

[28] Asher D.R., Thapa K., Dharia S.D., Khan N., Potter R.A., Rodino-Klapac L.R., Mendell J.R. (2020). Clinical development on the frontier: gene therapy for duchenne muscular dystrophy. Expert Opin. Biol. Ther..

[29] Duan D. (2018). Systemic AAV Micro-dystrophin Gene Therapy for Duchenne Muscular Dystrophy. Mol. Ther..

[30] Bagher P., Duan D., Segal S.S. (2011). Evidence for impaired neurovascular transmission in a murine model of Duchenne muscular dystrophy. J. Appl. Physiol. (1985).

[31] Laporte J., Hu L.J., Kretz C., Mandel J.L., Kioschis P., Coy J.F., Klauck S.M., Poustka A., Dahl N. (1996). A gene mutated in X-linked myotubular myopathy defines a new putative tyrosine phosphatase family conserved in yeast. Nat. Genet..

[32] Buj-Bello A., Laugel V., Messaddeq N., Zahreddine H., Laporte J., Pellissier J.F., Mandel J.L. (2002). The lipid phosphatase myotubularin is essential for skeletal muscle maintenance but not for myogenesis in mice. Proc. Natl. Acad. Sci. USA.

[33] Al-Qusairi L., Weiss N., Toussaint A., Berbey C., Messaddeq N., Kretz C., Sanoudou D., Beggs A.H., Allard B., Mandel J.L. (2009). T-tubule disorganization and defective excitation-contraction coupling in muscle fibers lacking myotubularin lipid phosphatase. Proc. Natl. Acad. Sci. USA.

[34] Beggs A.H., Byrne B.J., De Chastonay S., Haselkorn T., Hughes I., James E.S., Kuntz N.L., Simon J., Swanson L.C., Yang M.L. (2018). A multicenter, retrospective medical record review of X-linked myotubular myopathy: The recensus study. Muscle Nerve.

[35] Joubert R., Vignaud A., Le M., Moal C., Messaddeq N., Buj-Bello A. (2013). Site-specific Mtm1 mutagenesis by an AAV-Cre vector reveals that myotubularin is essential in adult muscle. Hum. Mol. Genet..

[36] Buj-Bello A., Fougerousse F., Schwab Y., Messaddeq N., Spehner D., Pierson C.R., Durand M., Kretz C., Danos O., Douar A.M. (2008). AAV-mediated intramuscular delivery of myotubularin corrects the myotubular myopathy phenotype in targeted murine muscle and suggests a function in plasma membrane homeostasis. Hum. Mol. Genet..

[37] Childers M.K., Joubert R., Poulard K., Moal C., Grange R.W., Doering J.A., Lawlor M.W., Rider B.E., Jamet T., Danièle N. (2014). Gene therapy prolongs survival and restores function in murine and canine models of myotubular myopathy. Sci. Transl. Med..

[38] Mack D.L., Poulard K., Goddard M.A., Latournerie V., Snyder J.M., Grange R.W., Elverman M.R., Denard J., Veron P., Buscara L. (2017). Systemic AAV8-Mediated Gene Therapy Drives Whole-Body Correction of Myotubular Myopathy in Dogs. Mol. Ther..

[39] Elverman M., Goddard M.A., Mack D., Snyder J.M., Lawlor M.W., Meng H., Beggs A.H., Buj-Bello A., Poulard K., Marsh A.P. (2017). Long-term effects of systemic gene therapy in a canine model of myotubular myopathy. Muscle Nerve.

[40] Annoussamy M., Lilien C., Gidaro T., Gargaun E., Chê V., Schara U., Gangfuß A., D’Amico A., Dowling J.J., Darras B.T. (2019). X-linked myotubular myopathy: A prospective international natural history study. Neurology.

[41] Raikwar S.P., Thangavel R., Dubova I., Ahmed M.E., Selvakumar P.G., Kempuraj D., Zaheer S., Iyer S., Zaheer A. (2018). Neuro-Immuno-Gene- and Genome-Editing-Therapy for Alzheimer’s Disease: Are We There Yet?. J. Alzheimers Dis..

[42] Koliatsos V.E., Nauta H.J., Clatterbuck R.E., Holtzman D.M., Mobley W.C., Price D.L. (1990). Mouse nerve growth factor prevents degeneration of axotomized basal forebrain cholinergic neurons in the monkey. J. Neurosci..

[43] Fischer W., Wictorin K., Björklund A., Williams L.R., Varon S., Gage F.H. (1987). Amelioration of cholinergic neuron atrophy and spatial memory impairment in aged rats by nerve growth factor. Nature.

[44] Rafii M.S., Baumann T.L., Bakay R.A., Ostrove J.M., Siffert J., Fleisher A.S., Herzog C.D., Barba D., Pay M., Salmon D.P. (2014). A phase1 study of stereotactic gene delivery of AAV2-NGF for Alzheimer’s disease. Alzheimers Dement..

[45] Rafii M.S., Tuszynski M.H., Thomas R.G., Barba D., Brewer J.B., Rissman R.A., Siffert J., Aisen P.S., AAV2-NGF Study Team (2018). Adeno-Associated Viral Vector (Serotype 2)-Nerve Growth Factor for Patients With Alzheimer Disease: A Randomized Clinical Trial. JAMA Neurol..

[46] Kaplitt M.G., Feigin A., Tang C., Fitzsimons H.L., Mattis P., Lawlor P.A., Bland R.J., Young D., Strybing K., Eidelberg D., During M.J. (2007). Safety and tolerability of gene therapy with an adeno-associated virus (AAV) borne GAD gene for Parkinson’s disease: an open label, phase I trial. Lancet.

[47] LeWitt P.A., Rezai A.R., Leehey M.A., Ojemann S.G., Flaherty A.W., Eskandar E.N., Kostyk S.K., Thomas K., Sarkar A., Siddiqui M.S. (2011). AAV2-GAD gene therapy for advanced Parkinson’s disease: a double-blind, sham-surgery controlled, randomised trial. Lancet Neurol..

[48] Niethammer M., Tang C.C., LeWitt P.A., Rezai A.R., Leehey M.A., Ojemann S.G., Flaherty A.W., Eskandar E.N., Kostyk S.K., Sarkar A. (2017). Long-term follow-up of a randomized AAV2-*GAD* gene therapy trial for Parkinson’s disease. JCI Insight.

[49] Niethammer M., Tang C.C., Vo A., Nguyen N., Spetsieris P., Dhawan V., Ma Y., Small M., Feigin A., During M.J. (2018). Gene therapy reduces Parkinson’s disease symptoms by reorganizing functional brain connectivity. Sci. Transl. Med..

[50] Eberling J.L., Jagust W.J., Christine C.W., Starr P., Larson P., Bankiewicz K.S., Aminoff M.J. (2008). Results from a phase I safety trial of hAADC gene therapy for Parkinson disease. Neurology.

[51] Christine C.W., Starr P.A., Larson P.S., Eberling J.L., Jagust W.J., Hawkins R.A., VanBrocklin H.F., Wright J.F., Bankiewicz K.S., Aminoff M.J. (2009). Safety and tolerability of putaminal AADC gene therapy for Parkinson disease. Neurology.

[52] Muramatsu S., Fujimoto K., Kato S., Mizukami H., Asari S., Ikeguchi K., Kawakami T., Urabe M., Kume A., Sato T. (2010). A phase I study of aromatic L-amino acid decarboxylase gene therapy for Parkinson’s disease. Mol. Ther..

[53] Christine C.W., Bankiewicz K.S., Van Laar A.D., Richardson R.M., Ravina B., Kells A.P., Boot B., Martin A.J., Nutt J., Thompson M.E., Larson P.S. (2019). Magnetic resonance imaging-guided phase 1 trial of putaminal AADC gene therapy for Parkinson’s disease. Ann. Neurol..

[54] Leone P., Shera D., McPhee S.W., Francis J.S., Kolodny E.H., Bilaniuk L.T., Wang D.J., Assadi M., Goldfarb O., Goldman H.W. (2012). Long-term follow-up after gene therapy for canavan disease. Sci. Transl. Med..

[55] Leone P., Janson C.G., Bilaniuk L., Wang Z., Sorgi F., Huang L., Matalon R., Kaul R., Zeng Z., Freese A. (2000). Aspartoacylase gene transfer to the mammalian central nervous system with therapeutic implications for Canavan disease. Ann. Neurol..

[56] Janson C., McPhee S., Bilaniuk L., Haselgrove J., Testaiuti M., Freese A., Wang D.J., Shera D., Hurh P., Rupin J. (2002). Clinical protocol. Gene therapy of Canavan disease: AAV-2 vector for neurosurgical delivery of aspartoacylase gene (ASPA) to the human brain. Hum. Gene Ther..

[57] von Jonquieres G., Spencer Z.H.T., Rowlands B.D., Klugmann C.B., Bongers A., Harasta A.E., Parley K.E., Cederholm J., Teahan O., Pickford R. (2018). Uncoupling N-acetylaspartate from brain pathology: implications for Canavan disease gene therapy. Acta Neuropathol..

[58] Hwu W.L., Chien Y.H., Lee N.C., Li M.H. (2018). Natural History of Aromatic L-Amino Acid Decarboxylase Deficiency in Taiwan. JIMD Rep..

[59] Hwu W.L., Muramatsu S., Tseng S.H., Tzen K.Y., Lee N.C., Chien Y.H., Snyder R.O., Byrne B.J., Tai C.H., Wu R.M. (2012). Gene therapy for aromatic L-amino acid decarboxylase deficiency. Sci. Transl. Med..

[60] Chien Y.-H., Lee N.-C., Tseng S.-H., Tai C.-H., Muramatsu S.I., Byrne B.J., Hwu W.L. (2017). Efficacy and safety of AAV2 gene therapy in children with aromatic L-amino acid decarboxylase deficiency: an open-label, phase 1/2 trial. Lancet Child Adolesc. Health.

[61] Kojima K., Nakajima T., Taga N., Miyauchi A., Kato M., Matsumoto A., Ikeda T., Nakamura K., Kubota T., Mizukami H. (2019). Gene therapy improves motor and mental function of aromatic l-amino acid decarboxylase deficiency. Brain.

[62] Echaniz-Laguna A., Cuisset J.M., Guyant-Marechal L., Aubourg P., Kremer L., Baaloul N., Verloes A., Beladgham K., Perrot J., Francou B., Latour P. (2020). Giant axonal neuropathy: a multicenter retrospective study with genotypic spectrum expansion. Neurogenetics.

[63] Bailey R.M., Armao D., Nagabhushan Kalburgi S., Gray S.J. (2018). Development of Intrathecal AAV9 Gene Therapy for Giant Axonal Neuropathy. Mol. Ther. Methods Clin. Dev..

[64] Su X., Kells A.P., Salegio E.A., Salegio E.A., Richardson R.M., Hadaczek P., Beyer J., Bringas J., Pivirotto P., Forsayeth J., Bankiewicz K.S. (2010). Real-time MR imaging with Gadoteridol predicts distribution of transgenes after convection-enhanced delivery of AAV2 vectors. Mol. Ther..

[65] Richardson R.M., Gimenez F., Salegio E.A., Su X., Bringas J., Berger M.S., Bankiewicz K.S. (2011). T2 imaging in monitoring of intraparenchymal real-time convection-enhanced delivery. Neurosurgery.

[66] San Sebastian W., Richardson R.M., Kells A.P., Lamarre C., Bringas J., Pivirotto P., Salegio E.A., Dearmond S.J., Forsayeth J., Bankiewicz K.S. (2012). Safety and tolerability of magnetic resonance imaging-guided convection-enhanced delivery of AAV2-hAADC with a novel delivery platform in nonhuman primate striatum. Hum. Gene Ther..

[67] Gray S.J., Matagne V., Bachaboina L., Yadav S., Ojeda S.R., Samulski R.J. (2011). Preclinical differences of intravascular AAV9 delivery to neurons and glia: a comparative study of adult mice and nonhuman primates. Mol. Ther..

[68] Gray S.J., Nagabhushan Kalburgi S., McCown T.J., Jude Samulski R. (2013). Global CNS gene delivery and evasion of anti-AAV-neutralizing antibodies by intrathecal AAV administration in non-human primates. Gene Ther..

[69] Samaranch L., Salegio E.A., San Sebastian W., Kells A.P., Foust K.D., Bringas J.R., Lamarre C., Forsayeth J., Kaspar B.K., Bankiewicz K.S. (2012). Adeno-associated virus serotype 9 transduction in the central nervous system of nonhuman primates. Hum. Gene Ther..

[70] Bailey R.M., Rozenberg A., Gray S.J. (2020). Comparison of high-dose intracisterna magna and lumbar puncture intrathecal delivery of AAV9 in mice to treat neuropathies. Brain Res..

[71] Bainbridge J.W., Smith A.J., Barker S.S., Robbie S., Henderson R., Balaggan K., Viswanathan A., Holder G.E., Stockman A., Tyler N. (2008). Effect of gene therapy on visual function in Leber’s congenital amaurosis. N. Engl. J. Med..

[72] Maguire A.M., Simonelli F., Pierce E.A., Pugh E.N., Mingozzi F., Bennicelli J., Banfi S., Marshall K.A., Testa F., Surace E.M. (2008). Safety and efficacy of gene transfer for Leber’s congenital amaurosis. N. Engl. J. Med..

[73] Hauswirth W.W., Aleman T.S., Kaushal S., Cideciyan A.V., Schwartz S.B., Wang L., Conlon T.J., Boye S.L., Flotte T.R., Byrne B.J., Jacobson S.G. (2008). Treatment of leber congenital amaurosis due to RPE65 mutations by ocular subretinal injection of adeno-associated virus gene vector: short-term results of a phase I trial. Hum. Gene Ther..

[74] Cideciyan A.V., Aleman T.S., Boye S.L., Schwartz S.B., Kaushal S., Roman A.J., Pang J.J., Sumaroka A., Windsor E.A., Wilson J.M. (2008). Human gene therapy for RPE65 isomerase deficiency activates the retinoid cycle of vision but with slow rod kinetics. Proc. Natl. Acad. Sci. USA.

[75] Jacobson S.G., Cideciyan A.V., Ratnakaram R., Heon E., Schwartz S.B., Roman A.J., Peden M.C., Aleman T.S., Boye S.L., Sumaroka A. (2012). Gene therapy for leber congenital amaurosis caused by RPE65 mutations: safety and efficacy in 15 children and adults followed up to 3 years. Arch. Ophthalmol..

[76] Maguire A.M., Russell S., Wellman J.A., Chung D.C., Yu Z.F., Tillman A., Wittes J., Pappas J., Elci O., Marshall K.A. (2019). Efficacy, Safety, and Durability of Voretigene Neparvovec-rzyl in RPE65 Mutation-Associated Inherited Retinal Dystrophy: Results of Phase 1 and 3 Trials. Ophthalmology.

[77] Cideciyan A.V. (2010). Leber congenital amaurosis due to RPE65 mutations and its treatment with gene therapy. Prog. Retin. Eye Res..

[78] Pierce E.A., Bennett J. (2015). The Status of RPE65 Gene Therapy Trials: Safety and Efficacy. Cold Spring Harb. Perspect. Med..

[79] Black A., Vasireddy V., Chung D.C., Maguire A.M., Gaddameedi R., Tolmachova T., Seabra M., Bennett J. (2014). Adeno-associated virus 8-mediated gene therapy for choroideremia: preclinical studies in in vitro and in vivo models. J. Gene Med..

[80] Komáromy A.M., Alexander J.J., Rowlan J.S., Garcia M.M., Chiodo V.A., Kaya A., Tanaka J.C., Acland G.M., Hauswirth W.W., Aguirre G.D. (2010). Gene therapy rescues cone function in congenital achromatopsia. Hum. Mol. Genet..

[81] Ofri R., Averbukh E., Ezra-Elia R., Ross M., Honig H., Obolensky A., Rosov A., Hauswirth W.W., Gootwine E., Banin E. (2018). Six Years and Counting: Restoration of Photopic Retinal Function and Visual Behavior Following Gene Augmentation Therapy in a Sheep Model of *CNGA3* Achromatopsia. Hum. Gene Ther..

[82] Michalakis S., Mühlfriedel R., Tanimoto N., Krishnamoorthy V., Koch S., Fischer M.D., Becirovic E., Bai L., Huber G., Beck S.C. (2010). Restoration of cone vision in the CNGA3-/- mouse model of congenital complete lack of cone photoreceptor function. Mol. Ther..

[83] Carvalho L.S., Xu J., Pearson R.A., Smith A.J., Bainbridge J.W., Morris L.M., Fliesler S.J., Ding X.Q., Ali R.R. (2011). Long-term and age-dependent restoration of visual function in a mouse model of CNGB3-associated achromatopsia following gene therapy. Hum. Mol. Genet..

[84] Beltran W.A., Cideciyan A.V., Iwabe S., Swider M., Kosyk M.S., McDaid K., Martynyuk I., Ying G.S., Shaffer J., Deng W.T. (2015). Successful arrest of photoreceptor and vision loss expands the therapeutic window of retinal gene therapy to later stages of disease. Proc. Natl. Acad. Sci. USA.

[85] Cideciyan A.V., Sudharsan R., Dufour V.L., Massengill M.T., Iwabe S., Swider M., Lisi B., Sumaroka A., Marinho L.F., Appelbaum T. (2018). Mutation-independent rhodopsin gene therapy by knockdown and replacement with a single AAV vector. Proc. Natl. Acad. Sci. USA.

[86] Constable I.J., Pierce C.M., Lai C.M., Magno A.L., Degli-Esposti M.A., French M.A., McAllister I.L., Butler S., Barone S.B., Schwartz S.D. (2016). Phase 2a Randomized Clinical Trial: Safety and Post Hoc Analysis of Subretinal rAAV.sFLT-1 for Wet Age-related Macular Degeneration. EBioMedicine.

[87] Constable I.J., Lai C.M., Magno A.L., French M.A., Barone S.B., Schwartz S.D., Blumenkranz M.S., Degli-Esposti M.A., Rakoczy E.P. (2017). Gene Therapy in Neovascular Age-related Macular Degeneration: Three-Year Follow-up of a Phase 1 Randomized Dose Escalation Trial. Am. J. Ophthalmol..

[88] Heier J.S., Kherani S., Desai S., Dugel P., Kaushal S., Cheng S.H., Delacono C., Purvis A., Richards S., Le-Halpere A. (2017). Intravitreous injection of AAV2-sFLT01 in patients with advanced neovascular age-related macular degeneration: a phase 1, open-label trial. Lancet.

[89] Cukras C., Wiley H.E., Jeffrey B.G., Sen H.N., Turriff A., Zeng Y., Vijayasarathy C., Marangoni D., Ziccardi L., Kjellstrom S. (2018). Retinal AAV8-RS1 Gene Therapy for X-Linked Retinoschisis: Initial Findings from a Phase I/IIa Trial by Intravitreal Delivery. Mol. Ther..

[90] Fischer M.D., Ochakovski G.A., Beier B., Seitz I.P., Vaheb Y., Kortuem C., Reichel F.F.L., Kuehlewein L., Kahle N.A., Peters T. (2019). Efficacy and Safety of Retinal Gene Therapy Using Adeno-Associated Virus Vector for Patients With Choroideremia: A Randomized Clinical Trial. JAMA Ophthalmol..

[91] Fischer M.D., Ochakovski G.A., Beier B., Seitz I.P., Vaheb Y., Kortuem C., Reichel F.F.L., Kuehlewein L., Kahle N.A., Peters T. (2020). Changes in Retinal Sensitivity after Gene Therapy in Choroideremia. Retina.

[92] Fischer M.D., Michalakis S., Wilhelm B., Zobor D., Muehlfriedel R., Kohl S., Weisschuh N., Ochakovski G.A., Klein R., Schoen C. (2020). Safety and Vision Outcomes of Subretinal Gene Therapy Targeting Cone Photoreceptors in Achromatopsia: A Nonrandomized Controlled Trial. JAMA Ophthalmol..

[93] Niederkorn J.Y. (2002). Immune privilege in the anterior chamber of the eye. Crit. Rev. Immunol..

[94] Stein-Streilein J., Streilein J.W. (2002). Anterior chamber associated immune deviation (ACAID): regulation, biological relevance, and implications for therapy. Int. Rev. Immunol..

[95] Garafalo A.V., Cideciyan A.V., Héon E., Sheplock R., Pearson A., WeiYang Yu C., Sumaroka A., Aguirre G.D., Jacobson S.G. (2020). Progress in treating inherited retinal diseases: Early subretinal gene therapy clinical trials and candidates for future initiatives. Prog. Retin. Eye Res..

[96] Xue K., Groppe M., Salvetti A.P., MacLaren R.E. (2017). Technique of retinal gene therapy: delivery of viral vector into the subretinal space. Eye (Lond.).

[97] Vasconcelos H.M., Lujan B.J., Pennesi M.E., Yang P., Lauer A.K. (2020). Intraoperative optical coherence tomographic findings in patients undergoing subretinal gene therapy surgery. Int. J. Retina Vitreous.

[98] Davis J.L., Gregori N.Z., MacLaren R.E., Lam B.L. (2019). Surgical Technique for Subretinal Gene Therapy in Humans with Inherited Retinal Degeneration. Retina.

[99] Bainbridge J.W., Mehat M.S., Sundaram V., Robbie S.J., Barker S.E., Ripamonti C., Georgiadis A., Mowat F.M., Beattie S.G., Gardner P.J. (2015). Long-term effect of gene therapy on Leber’s congenital amaurosis. N. Engl. J. Med..

[100] Le Meur G., Lebranchu P., Billaud F., Adjali O., Schmitt S., Bézieau S., Péréon Y., Valabregue R., Ivan C., Darmon C. (2018). Safety and Long-Term Efficacy of AAV4 Gene Therapy in Patients with RPE65 Leber Congenital Amaurosis. Mol. Ther..

[101] Russell S., Bennett J., Wellman J.A., Chung D.C., Yu Z.F., Tillman A., Wittes J., Pappas J., Elci O., McCague S. (2017). Efficacy and safety of voretigene neparvovec (AAV2-hRPE65v2) in patients with RPE65-mediated inherited retinal dystrophy: a randomised, controlled, open-label, phase 3 trial. Lancet.

[102] MacLaren R.E., Groppe M., Barnard A.R., Cottriall C.L., Tolmachova T., Seymour L., Clark K.R., During M.J., Cremers F.P., Black G.C. (2014). Retinal gene therapy in patients with choroideremia: initial findings from a phase 1/2 clinical trial. Lancet.

[103] Song C., Dufour V.L., Cideciyan A.V., Ye G.J., Swider M., Newmark J., Timmers A.M., Robinson P.M., Knop D.R., Chulay J.D. (2020). Dose Range Finding Studies with Two RPGR Transgenes in a Canine Model of X-linked Retinitis Pigmentosa Treated with Subretinal Gene Therapy. Hum. Gene Ther..

[104] http://ir.agtc.com/index.php/news-releases/news-release-details/agtc-reports-positive-six-month-data-its-ongoing-phase-12.

[105] Liu Q., Tan G., Levenkova N., Li T., Pugh E.N., Rux J.J., Speicher D.W., Pierce E.A. (2007). The proteome of the mouse photoreceptor sensory cilium complex. Mol. Cell. Proteomics.

[106] Pang J., Cheng M., Haire S.E., Barker E., Planelles V., Blanks J.C. (2006). Efficiency of lentiviral transduction during development in normal and rd mice. Mol. Vis..

[107] Lipinski D.M., Barnard A.R., Charbel Issa P., Singh M.S., De Silva S.R., Trabalza A., Eleftheriadou I., Ellison S.M., Mazarakis N.D., MacLaren R.E. (2014). Vesicular stomatitis virus glycoprotein- and Venezuelan equine encephalitis virus-derived glycoprotein-pseudotyped lentivirus vectors differentially transduce corneal endothelium, trabecular meshwork, and human photoreceptors. Hum. Gene Ther..

[108] Dyka F.M., Boye S.L., Chiodo V.A., Hauswirth W.W., Boye S.E. (2014). Dual adeno-associated virus vectors result in efficient in vitro and in vivo expression of an oversized gene, MYO7A. Hum. Gene Ther. Methods.

[109] Trapani I., Colella P., Sommella A., Iodice C., Cesi G., de Simone S., Marrocco E., Rossi S., Giunti M., Palfi A. (2014). Effective delivery of large genes to the retina by dual AAV vectors. EMBO Mol. Med..

[110] Jacobson S.G., Cideciyan A.V., Peshenko I.V., Sumaroka A., Olshevskaya E.V., Cao L., Schwartz S.B., Roman A.J., Olivares M.B., Sadigh S. (2013). Determining consequences of retinal membrane guanylyl cyclase (RetGC1) deficiency in human Leber congenital amaurosis en route to therapy: residual cone-photoreceptor vision correlates with biochemical properties of the mutants. Hum. Mol. Genet..

[111] Bouzia Z., Georgiou M., Hull S., Robson A.G., Fujinami K., Rotsos T., Pontikos N., Arno G., Webster A.R., Hardcastle A.J. (2020). GUCY2D-Associated Leber Congenital Amaurosis: A Retrospective Natural History Study in Preparation for Trials of Novel Therapies. Am. J. Ophthalmol..

[112] Boye S.L., Choudhury S., Crosson S., Di Pasquale G., Afione S., Mellen R., Makal V., Calabro K.R., Fajardo D., Peterson J. (2020). Novel AAV44.9-Based Vectors Display Exceptional Characteristics for Retinal Gene Therapy. Mol. Ther..

[113] Olsen T.W., Feng X., Wabner K., Conston S.R., Sierra D.H., Folden D.V., Smith M.E., Cameron J.D. (2006). Cannulation of the suprachoroidal space: a novel drug delivery methodology to the posterior segment. Am. J. Ophthalmol..

[114] Patel S.R., Lin A.S., Edelhauser H.F., Prausnitz M.R. (2011). Suprachoroidal drug delivery to the back of the eye using hollow microneedles. Pharm. Res..

[115] Kim Y.C., Edelhauser H.F., Prausnitz M.R. (2014). Targeted delivery of antiglaucoma drugs to the supraciliary space using microneedles. Invest. Ophthalmol. Vis. Sci..

[116] Ding K., Shen J., Hafiz Z., Hackett S.F., Silva R.L.E., Khan M., Lorenc V.E., Chen D., Chadha R., Zhang M. (2019). AAV8-vectored suprachoroidal gene transfer produces widespread ocular transgene expression. J. Clin. Invest..

[117] Yiu G., Chung S.H., Mollhoff I.N., Nguyen U.T., Thomasy S.M., Yoo J., Taraborelli D., Noronha G. (2020). Suprachoroidal and Subretinal Injections of AAV Using Transscleral Microneedles for Retinal Gene Delivery in Nonhuman Primates. Mol. Ther. Methods Clin. Dev..

[118] Kansara V., Muya L., Wan C.R., Ciulla T.A. (2020). Suprachoroidal Delivery of Viral and Nonviral Gene Therapy for Retinal Diseases. J. Ocul. Pharmacol. Ther..

[119] de Smet M.D., Lynch J.L., Dejneka N.S., Keane M., Khan I.J. (2018). A Subretinal Cell Delivery Method via Suprachoroidal Access in Minipigs: Safety and Surgical Outcomes. Invest. Ophthalmol. Vis. Sci..

[120] Kotterman M.A., Yin L., Strazzeri J.M., Flannery J.G., Merigan W.H., Schaffer D.V. (2015). Antibody neutralization poses a barrier to intravitreal adeno-associated viral vector gene delivery to non-human primates. Gene Ther..

[121] Yin L., Greenberg K., Hunter J.J., Dalkara D., Kolstad K.D., Masella B.D., Wolfe R., Visel M., Stone D., Libby R.T. (2011). Intravitreal injection of AAV2 transduces macaque inner retina. Invest. Ophthalmol. Vis. Sci..

[122] Dalkara D., Byrne L.C., Klimczak R.R., Visel M., Yin L., Merigan W.H., Flannery J.G., Schaffer D.V. (2013). In vivo-directed evolution of a new adeno-associated virus for therapeutic outer retinal gene delivery from the vitreous. Sci. Transl. Med..

[123] Ramachandran P.S., Lee V., Wei Z., Song J.Y., Casal G., Cronin T., Willett K., Huckfeldt R., Morgan J.I., Aleman T.S. (2017). Evaluation of Dose and Safety of AAV7m8 and AAV8BP2 in the Non-Human Primate Retina. Hum. Gene Ther..

[124] Byrne L.C., Day T.P., Visel M., Strazzeri J.A., Fortuny C., Dalkara D., Merigan W.H., Schaffer D.V., Flannery J.G. (2020). In vivo-directed evolution of adeno-associated virus in the primate retina. JCI Insight.

[125] Guy J., Feuer W.J., Davis J.L., Porciatti V., Gonzalez P.J., Koilkonda R.D., Yuan H., Hauswirth W.W., Lam B.L. (2017). Gene Therapy for Leber Hereditary Optic Neuropathy: Low- and Medium-Dose Visual Results. Ophthalmology.

[126] Feuer W.J., Schiffman J.C., Davis J.L., Porciatti V., Gonzalez P., Koilkonda R.D., Yuan H., Lalwani A., Lam B.L., Guy J. (2016). Gene Therapy for Leber Hereditary Optic Neuropathy: Initial Results. Ophthalmology.

[127] Byrne L.C., Oztürk B.E., Lee T., Fortuny C., Visel M., Dalkara D., Schaffer D.V., Flannery J.G. (2014). Retinoschisin gene therapy in photoreceptors, Müller glia or all retinal cells in the Rs1h-/- mouse. Gene Ther..

[128] Kay C.N., Ryals R.C., Aslanidi G.V., Min S.H., Ruan Q., Sun J., Dyka F.M., Kasuga D., Ayala A.E., Van Vliet K. (2013). Targeting photoreceptors via intravitreal delivery using novel, capsid-mutated AAV vectors. PLoS ONE.

[129] Li Q., Miller R., Han P.Y., Pang J., Dinculescu A., Chiodo V., Hauswirth W.W. (2008). Intraocular route of AAV2 vector administration defines humoral immune response and therapeutic potential. Mol. Vis..

[130] Ye G.J., Budzynski E., Sonnentag P., Miller P.E., Sharma A.K., Ver Hoeve J.N., Howard K., Knop D.R., Neuringer M., McGill T. (2015). Safety and Biodistribution Evaluation in Cynomolgus Macaques of rAAV2tYF-CB-hRS1, a Recombinant Adeno-Associated Virus Vector Expressing Retinoschisin. Hum. Gene Ther. Clin. Dev..

[131] Ye G.J., Budzynski E., Sonnentag P., Nork T.M., Miller P.E., Sharma A.K., Ver Hoeve J.N., Smith L.M., Arndt T., Calcedo R. (2016). Safety and Biodistribution Evaluation in Cynomolgus Macaques of rAAV2tYF-PR1.7-hCNGB3, a Recombinant AAV Vector for Treatment of Achromatopsia. Hum. Gene Ther. Clin. Dev..

[132] Vignal C., Uretsky S., Fitoussi S., Galy A., Blouin L., Girmens J.F., Bidot S., Thomasson N., Bouquet C., Valero S. (2018). Safety of rAAV2/2-ND4 Gene Therapy for Leber Hereditary Optic Neuropathy. Ophthalmology.

[133] Bouquet C., Vignal Clermont C., Galy A., Fitoussi S., Blouin L., Munk M.R., Valero S., Meunier S., Katz B., Sahel J.A., Thomasson N. (2019). Immune Response and Intraocular Inflammation in Patients With Leber Hereditary Optic Neuropathy Treated With Intravitreal Injection of Recombinant Adeno-Associated Virus 2 Carrying the ND4 Gene: A Secondary Analysis of a Phase 1/2 Clinical Trial. JAMA Ophthalmol..

[134] Maclachlan T.K., Lukason M., Collins M., Munger R., Isenberger E., Rogers C., Malatos S., Dufresne E., Morris J., Calcedo R. (2011). Preclinical safety evaluation of AAV2-sFLT01- a gene therapy for age-related macular degeneration. Mol. Ther..

[135] Adverum Biotechnologies (2020). Adverum Biotechnologies Announces Positive Interim Data from Cohorts 1-4 from OPTIC Phase 1 Trial of ADVM-022 Intravitreal Gene Therapy for wet AMD, Reports Recent Business Progress and Second Quarter 2020 Financial Results, August 10, 2020. https://investors.adverum.com/node/10426/pdf.

[136] Adverum Biotechnologies (2019). Adverum Biotechnologies Provides Clinical Program Update on ADVM-022 Gene Therapy for Wet AMD, April 15, 2019. https://investors.adverum.com/news-releases/news-release-details/adverum-biotechnologies-provides-clinical-program-update-advm.

[137] Nathwani A.C., Tuddenham E.G., Rangarajan S., Rosales C., McIntosh J., Linch D.C., Chowdary P., Riddell A., Pie A.J., Harrington C. (2011). Adenovirus-associated virus vector-mediated gene transfer in hemophilia B. N. Engl. J. Med..

[138] Nathwani A.C., Reiss U.M., Tuddenham E.G., Rosales C., Chowdary P., McIntosh J., Della Peruta M., Lheriteau E., Patel N., Raj D. (2014). Long-term safety and efficacy of factor IX gene therapy in hemophilia B. N. Engl. J. Med..

[139] Nathwani A.C., Reiss U., Tuddenham E., Chowdary P., McIntosh J., Riddell A., Pie J., Mahlangu J.N., Recht M., Shen Y.-M. (2018). Adeno-associated mediated gene transfer for hemophilia B: 8 year follow up and impact of removing” empty viral particles” on safety and efficacy of gene transfer. Blood.

[140] Chapin J., Rottensteiner H., Scheiflinger F., Monahan P.E. (2017). SHP648: an analysis of bleeding rates and Factor IX Consumption in the Phase I/II BAX 335 Gene Therapy Trial in Subjects with Hemophilia B. Res. Pract. Thromb. Haemost..

[141] Miesbach W., Meijer K., Coppens M., Kampmann P., Klamroth R., Schutgens R., Tangelder M., Castaman G., Schwäble J., Bonig H. (2018). Gene therapy with adeno-associated virus vector 5-human factor IX in adults with hemophilia B. Blood.

[142] Miesbach W., Meijer K., Coppens M.K., Kampmann M., Klamroth R., Schutgens R., Castaman G., Seifried E., Schwaeble J., Bönig H. (2019). Stable FIX Expression and Durable Reductions in Bleeding and Factor IX Consumption for up to 4 Years Following AMT-060 Gene Therapy in Adults with Severe or Moderate-Severe Hemophilia B. Blood.

[143] clinicaltrials.gov (2018). Safety and Dose Finding Study of DTX101 (AAVrh10FIX) in Adults With Moderate/Severe to Severe Hemophilia B. Last updated: November 14, 2018. NCT02618915. NCT02618915.

[144] George L.A. (2017). Hemophilia gene therapy comes of age. Hematology (Am. Soc. Hematol. Educ. Program).

[145] Chowdary P.S.S., Shapiro S., Makris M., Evans G., Boyce S., Talks K., Dolan G., Reiss U., Phillips M., Riddell A. (2020). A novel adeno associated virus (AAV) gene therapy (FLT180a) achieves normal FIX activity levels in severe Hemophilia B (HB) patients (B-AMAZE study). Res. Pract. Thromb. Haemost..

[146] George L.A., Sullivan S.K., Giermasz A., Rasko J.E.J., Samelson-Jones B.J., Ducore J., Cuker A., Sullivan L.M., Majumdar S., Teitel J. (2017). Hemophilia B Gene Therapy with a High-Specific-Activity Factor IX Variant. N. Engl. J. Med..

[147] George L.A., Sullivan S.K., Rasko J.E.J., Giermasz A., Samelson-Jones B.J., Ducore J.M., Teitel J.M., McGuinn C.E., Runowski A.R., Wright F. (2019). Efficacy and Safety in 15 Hemophilia B Patients Treated with the AAV Gene Therapy Vector Fidanacogene Elaparvovec and Followed for at Least 1 Year. Blood.

[148] Majowicz A.N.B., Bijmeijer B., Lampen M.H., Spronck L., de Haan M., Petry H. (2020). Therapeutic human Factor IX (hFIX) activity after single treatment with AMT-060 (AAV5-hFIX) or AMT-061 (AAV5-Padua hFIX) in Hemophilia B patients with pre-existing anti-AAV5 humoral immunity. Haemophilia.

[149] Rangarajan S., Walsh L., Lester W., Perry D., Madan B., Laffan M., Yu H., Vettermann C., Pierce G.F., Wong W.Y., Pasi K.J. (2017). AAV5-Factor VIII Gene Transfer in Severe Hemophilia A. N. Engl. J. Med..

[150] Pasi K.J., Rangarajan S., Mitchell N., Lester W., Symington E., Madan B., Laffan M., Russell C.B., Li M., Pierce G.F., Wong W.Y. (2020). Multiyear Follow-up of AAV5-hFVIII-SQ Gene Therapy for Hemophilia A. N. Engl. J. Med..

[151] BioMarin (2020). World Federation of Hemophilia Virtual Summit Update. First-in-human four-year follow-up study of durable therapeutic efficacy and safety: AAV gene therapy with Valoctocogene Roxaparvovec for severe hemophilia A, June 17, 2020.

[152] George L., Eyster E., Ragni M., Sullivan S., Samelson-Jones B., Evans M., MacDougall A., Curran M., Tompkins S., Wachtel K. (2020). Phase I/II Trial of SPK-8011: Stable and Durable FVIII Expression for >2 Years with Significant ABR Improvements in Initial Dose Cohorts Following AAV-Mediated FVIII Gene Transfer for Hemophilia A. Res. Pract. Thromb. Haemost..

[153] Konkle B.A., Stine K., Visweshwar N., Harrington T.J., Leavitt A.D., Giermasz A., Arkin S., Di Russo G., Snyder A., Woolfson A., Rouy D. (2019). Updated Follow-up of the Alta Study, a Phase 1/2, Open-Label, Adaptive, Dose-Ranging Study to Assess the Safety and Tolerability of SB-525 Gene Therapy. Blood.

[154] Nathwani A.C., Tuddenham E., Chowdary T.E., McIntosh J., Lee D., Rosales C., Phillips M., Pie J., Meagher M.M., Reiss U. (2018). GO-8: Preliminary Results of a Phase I/II Dose Escalation Trial of Gene Therapy for Haemophilia a Using a Novel Human Factor VIII Variant. Blood.

[155] Pipe S.W., Hay C.R.M., Sheehan J.P., Lissitchkov T., Detering E., Ribeiro S., Vanevski K. (2020). First-in-Human Gene Therapy Study of AAVhu37 Capsid Vector Technology in Severe Hemophilia A: Safety and FVIII Activity Results. Mol. Ther..

[156] Clnicaltrials.gov (2020). Gene Therapy Trial for Platelet Derived Factor VIII Production in Hemophilia A. Last updated: May 6, 2020. NCT03818763. NCT03818763.

[157] Cantore A., Nair N., Della Valle P., Di Matteo M., Màtrai J., Sanvito F., Brombin C., Di Serio C., D’Angelo A., Chuah M. (2012). Hyperfunctional coagulation factor IX improves the efficacy of gene therapy in hemophilic mice. Blood.

[158] Cantore A., Ranzani M., Bartholomae C.C., Volpin M., Valle P.D., Sanvito F., Sergi L.S., Gallina P., Benedicenti F., Bellinger D. (2015). Liver-directed lentiviral gene therapy in a dog model of hemophilia B. Sci. Transl. Med..

[159] Du L.M., Nurden P., Nurden A.T., Nichols T.C., Bellinger D.A., Jensen E.S., Haberichter S.L., Merricks E., Raymer R.A., Fang J. (2013). Platelet-targeted gene therapy with human factor VIII establishes haemostasis in dogs with haemophilia A. Nat. Commun..

[160] Shi Q., Wilcox D.A., Fahs S.A., Weiler H., Wells C.W., Cooley B.C., Desai D., Morateck P.A., Gorski J., Montgomery R.R. (2006). Factor VIII ectopically targeted to platelets is therapeutic in hemophilia A with high-titer inhibitory antibodies. J. Clin. Invest..

[161] Greene T.K., Wang C., Hirsch J.D., Zhai L., Gewirtz J., Thornton M.A., Miao H.Z., Pipe S.W., Kaufman R.J., Camire R.M. (2010). In vivo efficacy of platelet-delivered, high specific activity factor VIII variants. Blood.

[162] Follenzi A., Benten D., Novikoff P., Faulkner L., Raut S., Gupta S. (2008). Transplanted endothelial cells repopulate the liver endothelium and correct the phenotype of hemophilia A mice. J. Clin. Invest..

[163] Nathwani A.C., Rosales C., McIntosh J., Rastegarlari G., Nathwani D., Raj D., Nawathe S., Waddington S.N., Bronson R., Jackson S. (2011). Long-term safety and efficacy following systemic administration of a self-complementary AAV vector encoding human FIX pseudotyped with serotype 5 and 8 capsid proteins. Mol. Ther..

[164] Nathwani A.C., Nienhuis A.W., Davidoff A.M. (2014). Our journey to successful gene therapy for hemophilia B. Hum. Gene Ther..

[165] Iorio A., Stonebraker J.S., Chambost H., Makris M., Coffin D., Herr C., Germini F., Data and Demographics Committee of the World Federation of Hemophilia (2019). Establishing the Prevalence and Prevalence at Birth of Hemophilia in Males: A Meta-analytic Approach Using National Registries. Ann. Intern. Med..

[166] Peyvandi F., Garagiola I., Young G. (2016). The past and future of haemophilia: diagnosis, treatments, and its complications. Lancet.

[167] Shahani T., Covens K., Lavend’homme R., Jazouli N., Sokal E., Peerlinck K., Jacquemin M. (2014). Human liver sinusoidal endothelial cells but not hepatocytes contain factor VIII. J. Thromb. Haemost..

[168] Everett L.A., Cleuren A.C., Khoriaty R.N., Ginsburg D. (2014). Murine coagulation factor VIII is synthesized in endothelial cells. Blood.

[169] Madeira C.L., Layman M.E., de Vera R.E., Fontes P.A., Ragni M.V. (2009). Extrahepatic factor VIII production in transplant recipient of hemophilia donor liver. Blood.

[170] Kurachi K., Davie E.W. (1982). Isolation and characterization of a cDNA coding for human factor IX. Proc. Natl. Acad. Sci. USA.

[171] Mannucci P.M., Tuddenham E.G. (2001). The hemophilias--from royal genes to gene therapy. N. Engl. J. Med..

[172] Manco-Johnson M.J., Abshire T.C., Shapiro A.D., Riske B., Hacker M.R., Kilcoyne R., Ingram J.D., Manco-Johnson M.L., Funk S., Jacobson L. (2007). Prophylaxis versus episodic treatment to prevent joint disease in boys with severe hemophilia. N. Engl. J. Med..

[173] Manco-Johnson M.J., Soucie J.M., Gill J.C., Joint Outcomes Committee of the Universal Data Collection, US Hemophilia Treatment Center Network (2017). Prophylaxis usage, bleeding rates, and joint outcomes of hemophilia, 1999 to 2010: a surveillance project. Blood.

[174] Schrijvers L.H., Beijlevelt-van der Zande M., Peters M., Lock J., Cnossen M.H., Schuurmans M.J., Fischer K. (2016). Adherence to prophylaxis and bleeding outcome in haemophilia: a multicentre study. Br. J. Haematol..

[175] Croteau S.E., Neufeld E.J. (2015). Transition considerations for extended half-life factor products. Haemophilia.

[176] ZOLGENSMA (2019). Highlights of Prescribing Information (Zolgensma). https://www.avexis.com/us/Content/pdf/prescribing_information.pdf.

[177] Feldman A.G., Parsons J.A., Dutmer C.M., Veerapandiyan A., Hafberg E., Maloney N., Mack C.L. (2020). Subacute Liver Failure Following Gene Replacement Therapy for Spinal Muscular Atrophy Type 1. J. Pediatr..

[178] Nault J.C., Datta S., Imbeaud S., Franconi A., Mallet M., Couchy G., Letouzé E., Pilati C., Verret B., Blanc J.F. (2015). Recurrent AAV2-related insertional mutagenesis in human hepatocellular carcinomas. Nat. Genet..

[179] Chandler R.J., LaFave M.C., Varshney G.K., Trivedi N.S., Carrillo-Carrasco N., Senac J.S., Wu W., Hoffmann V., Elkahloun A.G., Burgess S.M., Venditti C.P. (2015). Vector design influences hepatic genotoxicity after adeno-associated virus gene therapy. J. Clin. Invest..

[180] Nakai H., Montini E., Fuess S., Storm T.A., Grompe M., Kay M.A. (2003). AAV serotype 2 vectors preferentially integrate into active genes in mice. Nat. Genet..

[181] Mazepa M.A., Monahan P.E., Baker J.R., Riske B.K., Soucie J.M., US Hemophilia Treatment Center Network (2016). Men with severe hemophilia in the United States: birth cohort analysis of a large national database. Blood.

[182] Goodgame B., Shaheen N.J., Galanko J., El-Serag H.B. (2003). The risk of end stage liver disease and hepatocellular carcinoma among persons infected with hepatitis C virus: publication bias?. Am. J. Gastroenterol..

[183] Freeman A.J., Dore G.J., Law M.G., Thorpe M., Von Overbeck J., Lloyd A.R., Marinos G., Kaldor J.M. (2001). Estimating progression to cirrhosis in chronic hepatitis C virus infection. Hepatology.

[184] Lu M., Li J., Zhang T., Rupp L.B., Trudeau S., Holmberg S.D., Moorman A.C., Spradling P.R., Teshale E.H., Xu F. (2016). Serum Biomarkers Indicate Long-term Reduction in Liver Fibrosis in Patients With Sustained Virological Response to Treatment for HCV Infection. Clin. Gastroenterol. Hepatol..

[185] Thalappillil A., Ragni M.V., Comer D.M., Yabes J.G. (2019). Incidence and risk factors for hepatocellular cancer in individuals with haemophilia: A National Inpatient Sample Study. Haemophilia.

[186] George L.A., Ragni M.V., Rasko J.E.J., Raffini L.J., Samelson-Jones B.J., Ozelo M., Hazbon M., Runowski A.R., Wellman J.A., Wachtel K. (2020). Long-Term Follow-Up of the First in Human Intravascular Delivery of AAV for Gene Transfer: AAV2-hFIX16 for Severe Hemophilia B. Mol. Ther..

[187] Pittman D.D., Marquette K.A., Kaufman R.J. (1994). Role of the B domain for factor VIII and factor V expression and function. Blood.

[188] Lind P., Larsson K., Spira J., Sydow-Bäckman M., Almstedt A., Gray E., Sandberg H. (1995). Novel forms of B-domain-deleted recombinant factor VIII molecules. Construction and biochemical characterization. Eur. J. Biochem..

[189] Sandberg H., Almstedt A., Brandt J., Gray E., Holmquist L., Oswaldsson U., Sebring S., Mikaelsson M. (2001). Structural and functional characteristics of the B-domain-deleted recombinant factor VIII protein, r-VIII SQ. Thromb. Haemost..

[190] Pittman D.D., Alderman E.M., Tomkinson K.N., Wang J.H., Giles A.R., Kaufman R.J. (1993). Biochemical, immunological, and in vivo functional characterization of B-domain-deleted factor VIII. Blood.

[191] McIntosh J., Lenting P.J., Rosales C., Lee D., Rabbanian S., Raj D., Patel N., Tuddenham E.G., Christophe O.D., McVey J.H. (2013). Therapeutic levels of FVIII following a single peripheral vein administration of rAAV vector encoding a novel human factor VIII variant. Blood.

[192] Simioni P., Tormene D., Tognin G., Gavasso S., Bulato C., Iacobelli N.P., Finn J.D., Spiezia L., Radu C., Arruda V.R. (2009). X-linked thrombophilia with a mutant factor IX (factor IX Padua). N. Engl. J. Med..

[193] Samelson-Jones B.J., Finn J.D., George L.A., Camire R.M., Arruda V.R. (2019). Hyperactivity of factor IX Padua (R338L) depends on factor VIIIa cofactor activity. JCI Insight.

[194] Lusher J.M., Arkin S., Abildgaard C.F., Schwartz R.S., Kogenate Previously Untreated Patient Study Group (1993). Recombinant factor VIII for the treatment of previously untreated patients with hemophilia A. Safety, efficacy, and development of inhibitors. N. Engl. J. Med..

[195] Hay C.R., Palmer B., Chalmers E., Liesner R., Maclean R., Rangarajan S., Williams M., Collins P.W., United Kingdom Haemophilia Centre Doctors’ Organisation (UKHCDO) (2011). Incidence of factor VIII inhibitors throughout life in severe hemophilia A in the United Kingdom. Blood.

[196] Gouw S.C., van der Bom J.G., Ljung R., Escuriola C., Cid A.R., Claeyssens-Donadel S., van Geet C., Kenet G., Mäkipernaa A., Molinari A.C., PedNet and RODIN Study Group (2013). Factor VIII products and inhibitor development in severe hemophilia A. N. Engl. J. Med..

[197] Recht M., Nemes L., Matysiak M., Manco-Johnson M., Lusher J., Smith M., Mannucci P., Hay C., Abshire T., O’Brien A. (2009). Clinical evaluation of moroctocog alfa (AF-CC), a new generation of B-domain deleted recombinant factor VIII (BDDrFVIII) for treatment of haemophilia A: demonstration of safety, efficacy, and pharmacokinetic equivalence to full-length recombinant factor VIII. Haemophilia.

[198] Xi M., Makris M., Marcucci M., Santagostino E., Mannucci P.M., Iorio A. (2013). Inhibitor development in previously treated hemophilia A patients: a systematic review, meta-analysis, and meta-regression. J. Thromb. Haemost..

[199] Samelson-Jones B.J., Arruda V.R. (2018). Protein-Engineered Coagulation Factors for Hemophilia Gene Therapy. Mol. Ther. Methods Clin. Dev..

[200] Finn J.D., Ozelo M.C., Sabatino D.E., Franck H.W., Merricks E.P., Crudele J.M., Zhou S., Kazazian H.H., Lillicrap D., Nichols T.C., Arruda V.R. (2010). Eradication of neutralizing antibodies to factor VIII in canine hemophilia A after liver gene therapy. Blood.

[201] Crudele J.M., Finn J.D., Siner J.I., Martin N.B., Niemeyer G.P., Zhou S., Mingozzi F., Lothrop C.D., Arruda V.R. (2015). AAV liver expression of FIX-Padua prevents and eradicates FIX inhibitor without increasing thrombogenicity in hemophilia B dogs and mice. Blood.

[202] Finn J.D., Nichols T.C., Svoronos N., Merricks E.P., Bellenger D.A., Zhou S., Simioni P., High K.A., Arruda V.R. (2012). The efficacy and the risk of immunogenicity of FIX Padua (R338L) in hemophilia B dogs treated by AAV muscle gene therapy. Blood.

[203] Mingozzi F., Hasbrouck N.C., Basner-Tschakarjan E., Edmonson S.A., Hui D.J., Sabatino D.E., Zhou S., Wright J.F., Jiang H., Pierce G.F. (2007). Modulation of tolerance to the transgene product in a nonhuman primate model of AAV-mediated gene transfer to liver. Blood.

[204] Ragni M.V., George L.A., Members of Working Group, (2019). The national blueprint for future factor VIII inhibitor clinical trials: NHLBI State of the Science (SOS) Workshop on factor VIII inhibitors. Haemophilia.

[205] Doerfler P.A., Nayak S., Corti M., Morel L., Herzog R.W., Byrne B.J. (2016). Targeted approaches to induce immune tolerance for Pompe disease therapy. Mol. Ther. Methods Clin. Dev..

[206] Rietveld I.M., Lijfering W.M., le Cessie S., Bos M.H.A., Rosendaal F.R., Reitsma P.H., Cannegieter S.C. (2019). High levels of coagulation factors and venous thrombosis risk: strongest association for factor VIII and von Willebrand factor. J. Thromb. Haemost..

[207] Koster T., Blann A.D., Briët E., Vandenbroucke J.P., Rosendaal F.R. (1995). Role of clotting factor VIII in effect of von Willebrand factor on occurrence of deep-vein thrombosis. Lancet.

[208] Kyrle P.A., Minar E., Hirschl M., Bialonczyk C., Stain M., Schneider B., Weltermann A., Speiser W., Lechner K., Eichinger S. (2000). High plasma levels of factor VIII and the risk of recurrent venous thromboembolism. N. Engl. J. Med..

[209] Thalji N.K., Ivanciu L., Davidson R., Gimotty P.A., Krishnaswamy S., Camire R.M. (2016). A rapid pro-hemostatic approach to overcome direct oral anticoagulants. Nat. Med..

[210] den Uijl I.E., Fischer K., Van Der Bom J.G., Grobbee D.E., Rosendaal F.R., Plug I. (2011). Analysis of low frequency bleeding data: the association of joint bleeds according to baseline FVIII activity levels. Haemophilia.

[211] Robinson M., George L.A., Samelson-Jones B.J., Arruda V.A., High K.A., Carr M.E., Tiefenbacher S. (2018). Activity of a FIX-Padua Transgene Product in Commonly Used FIX:C One-Stage and Chromogenic Assay Systems Following PF-06838435 (SPK-9001) Gene Delivery. Blood.

[212] Kitchen S., Jennings I., Makris M., Kitchen D.P., Woods T.A., Walker I.D. (2016). Factor VIII assay variability in postinfusion samples containing full length and B-domain deleted FVIII. Haemophilia.

[213] Zou C., Vercauteren K.O.A., Michailidis E., Kabbani M., Zoluthkin I., Quirk C., Chiriboga L., Yazicioglu M., Anguela X.M., Meuleman P. (2020). Experimental Variables that Affect Human Hepatocyte AAV Transduction in Liver Chimeric Mice. Mol. Ther. Methods Clin. Dev..

[214] Mingozzi F., Maus M.V., Hui D.J., Sabatino D.E., Murphy S.L., Rasko J.E., Ragni M.V., Manno C.S., Sommer J., Jiang H. (2007). CD8(+) T-cell responses to adeno-associated virus capsid in humans. Nat. Med..

[215] Faust S.M., Bell P., Cutler B.J., Ashley S.N., Zhu Y., Rabinowitz J.E., Wilson J.M. (2013). CpG-depleted adeno-associated virus vectors evade immune detection. J. Clin. Invest..

[216] clinicaltrials.gov (2020). Hemophilia B Gene Therapy With AAV8 Vector. Last updated March 12, 2019. NCT01620801. NCT01620801.

[217] Li C., Samulski R.J. (2020). Engineering adeno-associated virus vectors for gene therapy. Nat. Rev. Genet..

[218] Niemeyer G.P., Herzog R.W., Mount J., Arruda V.R., Tillson D.M., Hathcock J., van Ginkel F.W., High K.A., Lothrop C.D. (2009). Long-term correction of inhibitor-prone hemophilia B dogs treated with liver-directed AAV2-mediated factor IX gene therapy. Blood.

[219] Nguyen G.N., Everett J.K., Raymond H., Kafle S., Merricks E.P., Kazazian H.H., Nichols T.C., Bushman F.D., Sabatino D.E. (2019). Long-Term AAV-Mediated Factor VIII Expression in Nine Hemophilia A Dogs: A 10 Year Follow-up Analysis on Durability, Safety and Vector Integration. Blood.

[220] Lange A.M., Altynova E.S., Nguyen G.N., Sabatino D.E. (2016). Overexpression of factor VIII after AAV delivery is transiently associated with cellular stress in hemophilia A mice. Mol. Ther. Methods Clin. Dev..

[221] Zolotukhin I., Markusic D.M., Palaschak B., Hoffman B.E., Srikanthan M.A., Herzog R.W. (2016). Potential for cellular stress response to hepatic factor VIII expression from AAV vector. Mol. Ther. Methods Clin. Dev..

[222] Poothong J., Pottekat A., Siirin M., Campos A.R., Paton A.W., Paton J.C., Lagunas-Acosta J., Chen Z., Swift M., Volkmann N. (2020). Factor VIII exhibits chaperone-dependent and glucose-regulated reversible amyloid formation in the endoplasmic reticulum. Blood.

[223] Majowicz A., Nijmeijer B., Lampen M.H., Spronck L., de Haan M., Petry H., van Deventer S.J., Meyer C., Tangelder M., Ferreira V. (2019). Therapeutic hFIX Activity Achieved after Single AAV5-hFIX Treatment in Hemophilia B Patients and NHPs with Pre-existing Anti-AAV5 NABs. Mol. Ther. Methods Clin. Dev..

[224] Calcedo R., Wilson J.M. (2016). AAV Natural Infection Induces Broad Cross-Neutralizing Antibody Responses to Multiple AAV Serotypes in Chimpanzees. Hum. Gene Ther. Clin. Dev..

[225] Aronson S.J., Veron P., Collaud F., Hubert A., Delahais V., Honnet G., de Knegt R.J., Junge N., Baumann U., Di Giorgio A. (2019). Prevalence and Relevance of Pre-Existing Anti-Adeno-Associated Virus Immunity in the Context of Gene Therapy for Crigler-Najjar Syndrome. Hum. Gene Ther..

[226] Wang J., DeClercq J.J., Hayward S.B., Li P.W., Shivak D.A., Gregory P.D., Lee G., Holmes M.C. (2016). Highly efficient homology-driven genome editing in human T cells by combining zinc-finger nuclease mRNA and AAV6 donor delivery. Nucleic Acids Res..

[227] Mingozzi F., Chen Y., Murphy S.L., Edmonson S.C., Tai A., Price S.D., Metzger M.E., Zhou S., Wright J.F., Donahue R.E. (2012). Pharmacological modulation of humoral immunity in a nonhuman primate model of AAV gene transfer for hemophilia B. Mol. Ther..

[228] Wang L., Bell P., Somanathan S., Wang Q., He Z., Yu H., McMenamin D., Goode T., Calcedo R., Wilson J.M. (2015). Comparative Study of Liver Gene Transfer With AAV Vectors Based on Natural and Engineered AAV Capsids. Mol. Ther..

[229] (2018). Lysosomal storage disorders. Nat. Rev. Dis. Primers.

[230] Platt F.M., d’Azzo A., Davidson B.L., Neufeld E.F., Tifft C.J. (2018). Lysosomal storage diseases. Nat. Rev. Dis. Primers.

[231] Meikle P.J., Hopwood J.J., Clague A.E., Carey W.F. (1999). Prevalence of lysosomal storage disorders. JAMA.

[232] van der Meijden J.C., Kruijshaar M.E., Harlaar L., Rizopoulos D., van der Beek N.A.M.E., van der Ploeg A.T. (2018). Long-term follow-up of 17 patients with childhood Pompe disease treated with enzyme replacement therapy. J. Inherit. Metab. Dis..

[233] Sands M.S., Davidson B.L. (2006). Gene therapy for lysosomal storage diseases. Mol. Ther..

[234] Parenti G., Andria G., Ballabio A. (2015). Lysosomal storage diseases: from pathophysiology to therapy. Annu. Rev. Med..

[235] Raben N., Plotz P., Byrne B.J. (2002). Acid alpha-glucosidase deficiency (glycogenosis type II, Pompe disease). Curr. Mol. Med..

[236] Todd A.G., McElroy J.A., Grange R.W., Fuller D.D., Walter G.A., Byrne B.J., Falk D.J. (2015). Correcting Neuromuscular Deficits With Gene Therapy in Pompe Disease. Ann. Neurol..

[237] Byrne B.J., Falk D.J., Pacak C.A., Nayak S., Herzog R.W., Elder M.E., Collins S.W., Conlon T.J., Clement N., Cleaver B.D. (2011). Pompe disease gene therapy. Hum. Mol. Genet..

[238] Corti M., Liberati C., Smith B.K., Lawson L.A., Tuna I.S., Conlon T.J., Coleman K.E., Islam S., Herzog R.W., Fuller D.D. (2017). Safety of Intradiaphragmatic Delivery of Adeno-Associated Virus-Mediated Alpha-Glucosidase (rAAV1-CMV-hGAA) Gene Therapy in Children Affected by Pompe Disease. Hum. Gene Ther. Clin. Dev..

[239] Byrne P.I., Collins S., Mah C.C., Smith B., Conlon T., Martin S.D., Corti M., Cleaver B., Islam S., Lawson L.A. (2014). Phase I/II trial of diaphragm delivery of recombinant adeno-associated virus acid alpha-glucosidase (rAAaV1-CMV-GAA) gene vector in patients with Pompe disease. Hum. Gene Ther. Clin. Dev..

[240] Smith B.K., Collins S.W., Conlon T.J., Mah C.S., Lawson L.A., Martin A.D., Fuller D.D., Cleaver B.D., Clément N., Phillips D. (2013). Phase I/II trial of adeno-associated virus-mediated alpha-glucosidase gene therapy to the diaphragm for chronic respiratory failure in Pompe disease: initial safety and ventilatory outcomes. Hum. Gene Ther..

[241] Corti M., Elder M., Falk D., Lawson L., Smith B., Nayak S., Conlon T., Clément N., Erger K., Lavassani E. (2014). B-Cell Depletion is Protective Against Anti-AAV Capsid Immune Response: A Human Subject Case Study. Mol. Ther. Methods Clin. Dev..

[242] Corti M., Smith B.K., Falk D.J., Lawson L.A., Fuller D.D., Subramony S.H., Byrne B.J., Christou E.A. (2015). Altered activation of the tibialis anterior in individuals with Pompe disease: Implications for motor unit dysfunction. Muscle Nerve.

[243] BioSpace (2018). Audentes Therapeutics Reports Second Quarter 2018 Financial Results and Provides Update on ASPIRO, the Phase 1/2 Clinical Trial of AT132 in Patients with X-Linked Myotubular Myopathy. August 7, 2018. https://www.biospace.com/article/releases/audentes-therapeutics-reports-second-quarter-2018-financial-results-and-provides-update-on-aspiro-the-phase-1-2-clinical-trial-of-at132-in-patients-with-x-linked-myotubular-myopathy/.

[244] Asklepios BioPharmaceutical (2019). First Patient Dosed with Gene Therapy in Phase 1/2 Study of ACTUS-101 in Patients with Pompe Disease. January 22, 2019. https://www.askbio.com/first-patient-dosed-with-gene-therapy-in-phase-1-2-study-of-actus-101-in-patients-with-pompe-disease/.

[245] Stern G. (2014). Niemann-Pick’s and Gaucher’s diseases. Parkinsonism Relat. Disord..

[246] Dunbar C., Kohn D. (1996). Retroviral mediated transfer of the cDNA for human glucocerebrosidase into hematopoietic stem cells of patients with Gaucher disease. A phase I study. Hum. Gene Ther..

[247] Fink J.K., Correll P.H., Perry L.K., Brady R.O., Karlsson S. (1990). Correction of glucocerebrosidase deficiency after retroviral-mediated gene transfer into hematopoietic progenitor cells from patients with Gaucher disease. Proc. Natl. Acad. Sci. USA.

[248] Dunbar C.E., Kohn D.B., Schiffmann R., Barton N.W., Nolta J.A., Esplin J.A., Pensiero M., Long Z., Lockey C., Emmons R.V. (1998). Retroviral transfer of the glucocerebrosidase gene into CD34+ cells from patients with Gaucher disease: in vivo detection of transduced cells without myeloablation. Hum. Gene Ther..

[249] Dahl M., Doyle A., Olsson K., Månsson J.E., Marques A.R.A., Mirzaian M., Aerts J.M., Ehinger M., Rothe M., Modlich U. (2015). Lentiviral gene therapy using cellular promoters cures type 1 Gaucher disease in mice. Mol. Ther..

[250] Chan B., Adam D.N. (2018). A Review of Fabry Disease. Skin Therapy Lett..

[251] Felis A., Whitlow M., Kraus A., Warnock D.G., Wallace E. (2019). Current and Investigational Therapeutics for Fabry Disease. Kidney Int. Rep..

[252] Nicholls K., Bleasel K., Becker G. (2012). Severe infusion reactions to fabry enzyme replacement therapy: rechallenge after tracheostomy. JIMD Rep..

[253] Lenders M., Stypmann J., Duning T., Schmitz B., Brand S.M., Brand E. (2016). Serum-Mediated Inhibition of Enzyme Replacement Therapy in Fabry Disease. J. Am. Soc. Nephrol..

[254] Ohashi T. (2019). Gene therapy for lysosomal storage diseases and peroxisomal diseases. J. Hum. Genet..

[255] Huang J., Khan A., Au B.C., Barber D.L., López-Vásquez L., Prokopishyn N.L., Boutin M., Rothe M., Rip J.W., Abaoui M. (2017). Lentivector Iterations and Pre-Clinical Scale-Up/Toxicity Testing: Targeting Mobilized CD34^+^ Cells for Correction of Fabry Disease. Mol. Ther. Methods Clin. Dev..

[256] Sangamo Therapeutics (2019). Sangamo Announces FDA Acceptance of IND Application for ST-920 Gene Therapy Candidate for Fabry Disease. February 20, 2019. https://investor.sangamo.com/news-releases/news-release-details/sangamo-announces-fda-acceptance-ind-application-st-920-gene.

[257] Freeline Therapeutics (2020). Freeline receives Orphan Drug Designation from the European Commission for FLT190 for the treatment of Fabry Disease. May 4, 2020. https://www.freeline.life/investors-media/newsroom/freeline-receives-orphan-drug-designation-from-the-fda-for-flt190-for-the-treatment-of-fabry-disease/.

[258] Tardieu M., Zérah M., Husson B., de Bournonville S., Deiva K., Adamsbaum C., Vincent F., Hocquemiller M., Broissand C., Furlan V. (2014). Intracerebral administration of adeno-associated viral vector serotype rh.10 carrying human SGSH and SUMF1 cDNAs in children with mucopolysaccharidosis type IIIA disease: results of a phase I/II trial. Hum. Gene Ther..

[259] Tardieu M., Zérah M., Gougeon M.L., Ausseil J., de Bournonville S., Husson B., Zafeiriou D., Parenti G., Bourget P., Poirier B. (2017). Intracerebral gene therapy in children with mucopolysaccharidosis type IIIB syndrome: an uncontrolled phase 1/2 clinical trial. Lancet Neurol..

[260] Butz E.S., Chandrachud U., Mole S.E., Cotman S.L. (2020). Moving towards a new era of genomics in the neuronal ceroid lipofuscinoses. Biochim. Biophys. Acta Mol. Basis Dis..

[261] Nelvagal H.R., Lange J., Takahashi K., Tarczyluk-Wells M.A., Cooper J.D. (2020). Pathomechanisms in the neuronal ceroid lipofuscinoses. Biochim. Biophys. Acta Mol. Basis Dis..

[262] Schulz A., Kohlschütter A., Mink J., Simonati A., Williams R. (2013). NCL diseases - clinical perspectives. Biochim. Biophys. Acta.

[263] Worgall S., Sondhi D., Hackett N.R., Kosofsky B., Kekatpure M.V., Neyzi N., Dyke J.P., Ballon D., Heier L., Greenwald B.M. (2008). Treatment of late infantile neuronal ceroid lipofuscinosis by CNS administration of a serotype 2 adeno-associated virus expressing CLN2 cDNA. Hum. Gene Ther..

[264] Cain J.T., Likhite S., White K.A., Timm D.J., Davis S.S., Johnson T.B., Dennys-Rivers C.N., Rinaldi F., Motti D., Corcoran S. (2019). Gene Therapy Corrects Brain and Behavioral Pathologies in CLN6-Batten Disease. Mol. Ther..

